# Molecular architecture of the *C*. *elegans* centriole

**DOI:** 10.1371/journal.pbio.3001784

**Published:** 2022-09-15

**Authors:** Alexander Woglar, Marie Pierron, Fabian Zacharias Schneider, Keshav Jha, Coralie Busso, Pierre Gönczy

**Affiliations:** Swiss Institute for Experimental Cancer Research (ISREC), School of Life Sciences, Swiss Federal Institute of Technology Lausanne (EPFL), Lausanne, Switzerland; Institut Curie, FRANCE

## Abstract

Uncovering organizing principles of organelle assembly is a fundamental pursuit in the life sciences. *Caenorhabditis elegans* was key in identifying evolutionary conserved components governing assembly of the centriole organelle. However, localizing these components with high precision has been hampered by the minute size of the worm centriole, thus impeding understanding of underlying assembly mechanisms. Here, we used Ultrastructure Expansion coupled with STimulated Emission Depletion (U-Ex-STED) microscopy, as well as electron microscopy (EM) and electron tomography (ET), to decipher the molecular architecture of the worm centriole. Achieving an effective lateral resolution of approximately 14 nm, we localize centriolar and PeriCentriolar Material (PCM) components in a comprehensive manner with utmost spatial precision. We found that all 12 components analysed exhibit a ring-like distribution with distinct diameters and often with a 9-fold radial symmetry. Moreover, we uncovered that the procentriole assembles at a location on the centriole margin where SPD-2 and ZYG-1 also accumulate. Moreover, SAS-6 and SAS-5 were found to be present in the nascent procentriole, with SAS-4 and microtubules recruited thereafter. We registered U-Ex-STED and EM data using the radial array of microtubules, thus allowing us to map each centriolar and PCM protein to a specific ultrastructural compartment. Importantly, we discovered that SAS-6 and SAS-4 exhibit a radial symmetry that is offset relative to microtubules, leading to a chiral centriole ensemble. Furthermore, we established that the centriole is surrounded by a region from which ribosomes are excluded and to which SAS-7 localizes. Overall, our work uncovers the molecular architecture of the *C*. *elegans* centriole in unprecedented detail and establishes a comprehensive framework for understanding mechanisms of organelle biogenesis and function.

## Abstract 2

The nematode Caenorhabditis elegans was key in identifying components involved in centriole assembly, but the small size of the worm centriole has hampered the localization of those components. This study uses a combination of U-Ex-STED microscopy, electron microscopy and electron tomography to reveal the molecular architecture of the C. elegans centriole in unprecedented detail.

## Introduction

Centrioles are membrane-less organelles that were present in the last common ancestor of eukaryotes (reviewed in [1]). In cells with flagella or cilia, centrioles act as basal bodies that template the formation of these structures. Moreover, in animal cells, centrioles form the core of the centrosome, which organizes microtubules and is thereby critical for fundamental cellular processes, including polarity and division (reviewed in [2]). In most organisms, centrioles are cylindrical organelles approximately 500 nm high and 250 nm wide, with a 9-fold radially symmetric distribution of microtubules (reviewed in [3,4]). Centriolar microtubules are usually organized in triplets in the proximal region of the organelle and in doublets more distally. Triplet and doublet microtubules are twisted in a clockwise direction with respect to the microtubules when viewed from the distal end of the centriole, resulting in the characteristic chiral geometry of the organelle. This 9-fold radially symmetric architecture is also imparted onto the ciliary and flagellar axoneme that stem from centriolar microtubules and might be evolutionarily conserved because it provides an optimal geometry for axonemal motility. Despite important progress in recent years, the detailed molecular architecture of the centriole, including the root of its characteristic chirality, remains incompletely understood.

There are variations in the architectural features of centrioles in some systems, which are usually correlated with the absence or reduction of ciliary and flagellar motility (reviewed in [5]). For instance, in the nematode *Caenorhabditis elegans*, motile cilia and flagella are absent, and the sperm moves in an amoeboid fashion. Perhaps in the absence of evolutionary pressure for ciliary and flagellar motility, centrioles are smaller (approximately 175 nm high and 120 nm wide) in the embryo [6–8] and comprise a radial arrangement of 9 microtubule singlets instead of the usual triplets and doublets [9]. Electron microscopy (EM) of centrioles in the *C*. *elegan*s embryo revealed ultrastructural compartments besides microtubules, including 9 peripheral paddlewheels, as well as the central tube and, more centrally still, the inner tube [6–8]. EM analysis of embryonic centrioles also led to the notion that each paddlewheel is offset with respect to its accompanying microtubule, with a clockwise twist when viewed from the distal end, resulting in a chiral ensemble [8]. Whether chirality of the *C*. *elegans* centriole is apparent more centrally in the organelle, where the assembly process is thought to initiate, is not known.

As in other systems, starting approximately at the onset of S phase, the 2 resident centrioles in *C*. *elegans* each seed the assembly of a procentriole in their vicinity, such that 4 centriolar units are present during mitosis, 2 per spindle pole. Comprehensive genetic and functional genomic screens conducted in *C*. *elegans* led to the discovery of 6 components essential for procentriole formation (reviewed in [10–12]). Molecular epistasis experiments uncovered the order in which these proteins are recruited to the worm organelle [7,13]. These experiments established that SAS-7 and SPD-2 (Cep192 in humans) are first recruited to the resident centriole. Thereafter, the kinase ZYG-1 (Plk4 in humans) directs the interacting coiled-coil proteins SAS-6 (HsSAS-6 in humans) and SAS-5 (STIL in humans) to the procentriole assembly site. This is followed by SAS-4 (CPAP in human) recruitment to the procentriole, a protein thought to enable the addition of microtubules to the SAS-6/SAS-5 scaffold. Relatives of SPD-2, ZYG-1, SAS-6, SAS-5, and SAS-4 in other systems are recruited in a similar sequence and exert analogous functions in procentriole formation (reviewed in [10–12]).

SAS-6 is the main building block of a scaffold referred to as the cartwheel, which is thought to contribute to imparting the 9-fold radial symmetry of the organelle (reviewed in [14,15]). Whereas SAS-6 proteins in other systems self-assemble into ring-containing polymers that stack to form the cartwheel, structural and biophysical evidence obtained with the *C*. *elegans* protein has led to the suggestion that SAS-6 forms a steep spiral [16]. However, whether this is the case in vivo has not been addressed.

HYLS-1 and SAS-1 are 2 additional *C*. *elegans* centriolar proteins, which are dispensable for procentriole assembly. However, HYLS-1 is needed for generating nonmotile cilia [17], whereas SAS-1 is critical for maintaining the integrity of the centriole once formed [18]. In addition, the Polo-like kinase PLK-1 is present at centrioles in the early worm embryo [19]. As in other systems, *C*. *elegans* centrioles recruit the PeriCentriolar Material (PCM), thus forming the centrosome, which acts as a microtubule organizing center (reviewed in [20]). Assembly of the *C*. *elegans* PCM core, which has been defined as the set of PCM proteins that are also present in interphase [21], relies on the interacting proteins SPD-2 [22,23] and SPD-5 [24], as well as on SAS-7 [8] and PCDM-1 [21]. Furthermore, the γ-tubulin protein TBG-1 [25,26], together with the γ-tubulin interacting proteins GIP-1 and GIP-2 [26], as well as the γ-tubulin partner MZT-1 [27] are present in the worm PCM core. Additional proteins, including PLK-1 and AIR-1 [28,29], as well as TAC-1 and ZYG-9 [30,31], are recruited to this PCM core when the centriole matures in mitosis in the embryo, leading to increased microtubule nucleation. Despite the probably near-comprehensive list of component parts of the centriole and the PCM core in *C*. *elegans*, the very small dimensions of the worm organelle have thus far prevented localizing with precision where each component resides, thus limiting understanding of how they function.

The molecular architecture of the centrioles has been investigated using 3D-Structured Illumination Microscopy (SIM) or STimulated Emission Depletion (STED) super-resolution microscopy in other systems where the organelle is larger than in *C*. *elegans*, including human cells and *Drosophila* [32–34]. Moreover, ultrastructure expansion (U-Ex) microscopy has been utilized to investigate the molecular architecture of centrioles from human cells [35,36]. In this method, the sample is embedded in a gel that is then expanded isotropically several fold, thus likewise expanding the effective resolution [37]. SIM, STED, and U-Ex have enabled placing in a more refined manner a subset of components in the centriole map in these systems. However, the resolution achieved with these approaches alone would be likely insufficient to resolve the molecular architecture of the minute worm centriole.

Here, we set out to map in a comprehensive manner and with utmost precision the distribution of centriolar as well as PCM core component in the gonad of *C*. *elegans*. Considering the very small size of the worm centriole, we combined U-Ex and STED, reaching an effective lateral resolution of approximately 14 nm. Using mainly endogenously tagged components and validated antibodies, we could thus determine with exquisite precision the localization of 12 centriolar and PCM core proteins. Of particular interest, this revealed that SAS-6 and SAS-4 exhibit an angular offset with respect to the microtubules, resulting in a chiral arrangement in the organelle center. Moreover, we acquired a large corresponding EM data set, which we overlaid with the U-Ex-STED images to map each centriolar protein to a specific ultrastructural compartment of the organelle. Overall, we uncovered the molecular architecture of the *C*. *elegans* centriole and provide an unprecedented framework for a mechanistic dissection of centriole assembly and function.

## Results

### Combining nuclei spreading and U-Ex microscopy for improved resolution of centrioles

**Fig 1:**
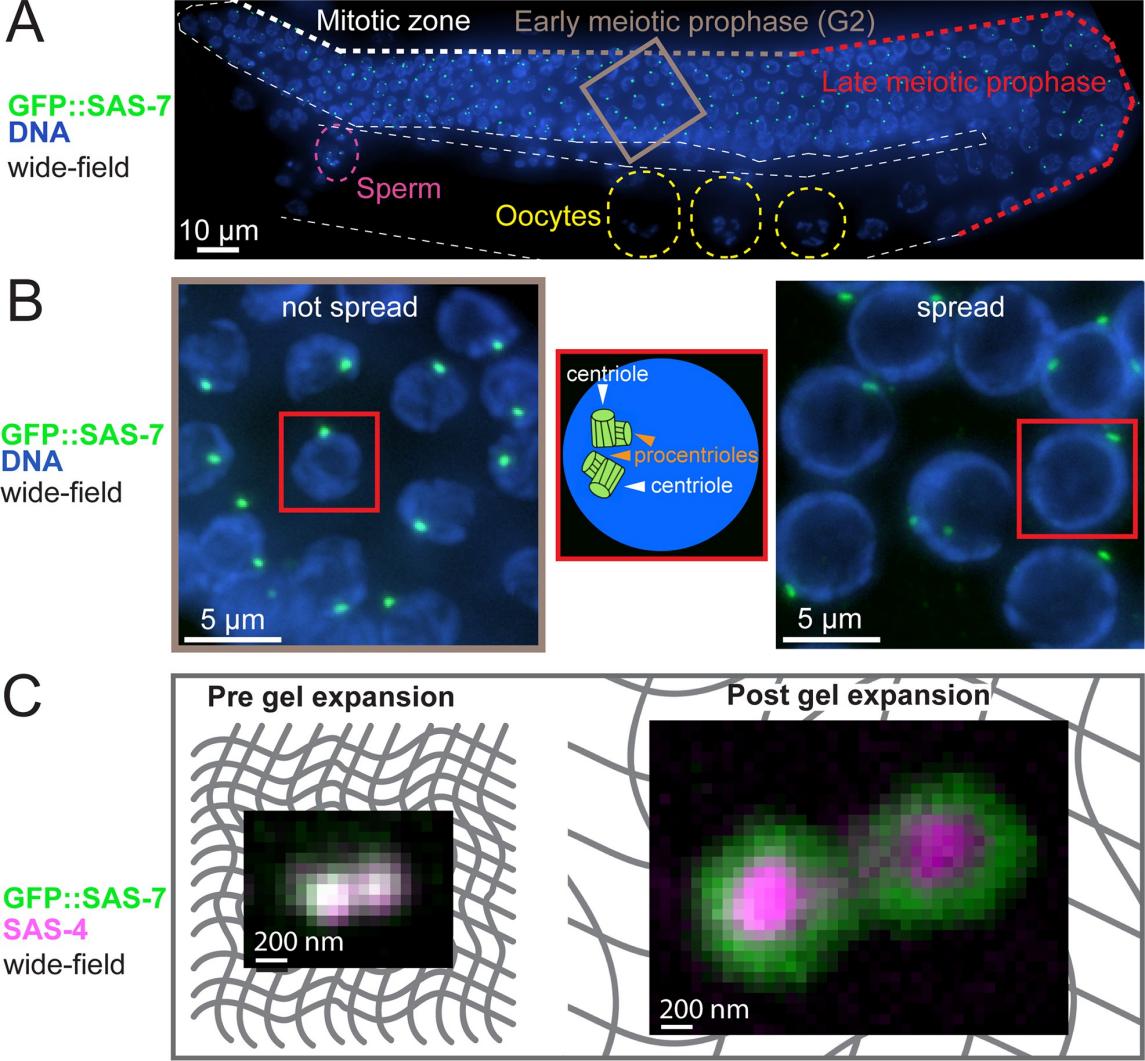
Combining gonad nuclei spreading and U-Ex microscopy to analyze worm gonad centrioles. (A) Widefield imaging of ethanol-fixed worm expressing GFP::SAS-7. One layer of nuclei of the gonad is max intensity Z-projected (in this case, a height of 6.25 μm). White, grey, and red bold dashed lines indicate progression through the gonad from the mitotic zone to early and then late meiotic prophase; other white dashed line outlines the gonad. Grey box is magnified in (B). Yellow dashed regions mark 3 oocytes, purple dashed region the spermatheca. Note that centrioles are eliminated in oocytes, prior to fertilization. (B) (Left) Magnification of grey box region from (A). (Middle) Schematic representation of a single nucleus shown in the left and right panels. (Right) Early prophase region of a spread gonad from a worm expressing GFP::SAS-7. Note that spread nuclei are flattened and thus occupy a larger area compared to not spread nuclei. Note also that at this stage, centrioles do not act as microtubule organizing centers [55,56]. (C) Widefield imaging of centrioles in the early prophase region of the gonad from worms expressing GFP::SAS-7 before (left) and after (right) gel expansion. Grey mesh in the background represents the gel matrix.

We set out to analyze the molecular architecture of the *C*. *elegans* centriole with utmost spatial resolution, using the adult hermaphrodite gonad as an experimental system (Fig 1A). The distal part of the syncytial gonad (the “mitotic zone” from here on) constitutes a stem cell pool where nuclei undergo cell cycles characterized by short G1 and M phases, with merely approximately 2% of nuclei being in one of these 2 phases combined [38]. Once nuclei have traveled far enough from the distal end of the gonad, they undergo premeiotic S phase and enter meiotic prophase I, a prolonged G2 phase during which meiotic recombination occurs.

The gonad can be easily extruded from the animal and contains hundreds of nuclei, which are almost all in S or G2 phases of the cell cycle. Since procentriole formation begins in early S phase, most gonad nuclei harbor 2 pairs of centriole/procentriole, which are in close proximity to one another and cannot be resolved by immunofluorescence (IF) in widefield microscopy, usually appearing instead as a single focus (Fig 1B, left). We took 2 steps to improve the spatial resolution for our analysis. First, nuclei from extracted gonads were adhered as a single layer to a coverslip using mild chromatin spreading [39], resulting in superior detection by IF since the specimen is closer to the coverslip. Moreover, the pool of cytoplasmic proteins, which would otherwise contribute to poor signal-to-noise ratio of the centriolar signal, is largely washed out in this manner (Fig 1B, right). Second, we adapted previously validated ultrastructure gel expansion methods (U-Ex) [35,37], reaching approximately 5-fold isotropic expansion of the specimens (Fig 1C, Materials and methods). Combination of spreading with U-ExM enabled us to distinguish centriole and procentriole with widefield microscopy (S1A Fig), as well as to localize components to distinct regions within the *C*. *elegans* centriole (Fig 1C, right).

### Procentriole assembly: Onset and maturation

**Fig 2:**
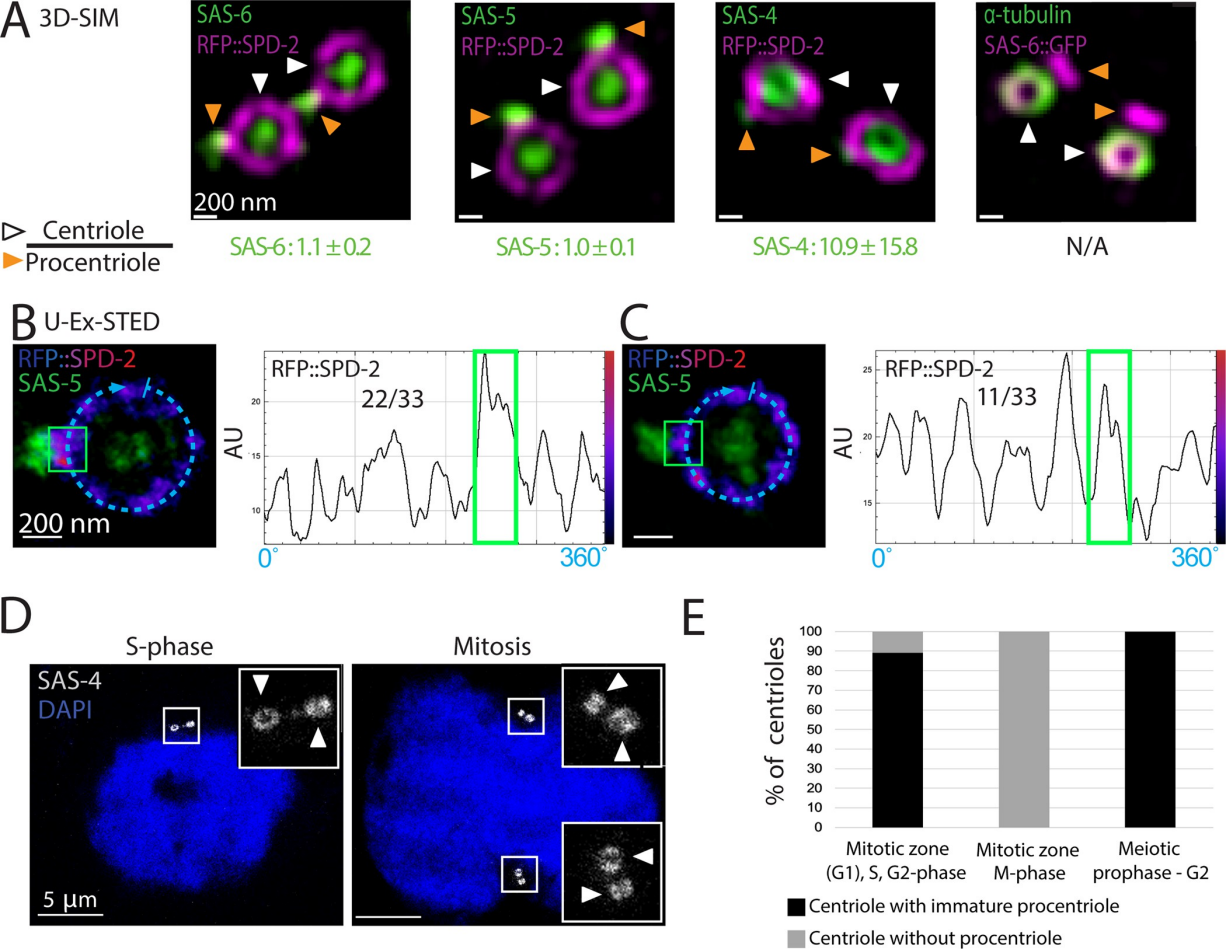
Composition and maturation of the procentriole. (A) 3D-SIM sum intensity Z-projected images of expanded centrioles from early meiotic prophase stained for the indicated proteins. White arrowheads point to centrioles, orange arrowheads to procentrioles. The presence of a ring-like distribution of RFP::SPD-2 (magenta) around one of the 2 green foci (SAS-6, SAS-5, or SAS-4) served to identify the centriole. The numbers below the images represent the ratio between the fluorescence of the indicated component in the centriole versus the procentriole (*N* = SAS-6: 8, SAS-5: 17, SAS-4: 18; ± indicates SD). Here and in all other figures, scale bars within a series represent the same length (e.g., 200 nm in this case). (B, C) (Left) U-Ex-STED of centrioles from early meiotic prophase revealing RFP::SPD-2 and SAS-5 distribution. RFP::SPD-2 signals are displayed with the LUT “Fire” (low intensities in blue, high intensities in magenta and red). (Right) Corresponding signal intensity profiles along the dashed line depicted in the image (10 pixels wide). The green boxes in the images and the graphs indicate RFP::SPD-2 located under the procentriole identified by SAS-5. In the majority of cases, the RFP::SPD-2 signal is wider and brighter below the procentriole than anywhere else in the centriole (B, 22/33), whereas such enrichment could not be detected in the remainder cases (C, 11/33). (D) U-Ex-STED of nuclei in either S-phase (left) or mitosis (right) from the mitotic zone of the gonad. White arrowheads point to centrioles fully decorated with SAS-4 (mature centrioles). SAS-4 signal intensity between the pair of centrioles present on each pole of the mitotic spindle varies by merely 14 ± 9% SD, *N* = 12 pairs. (E) Percentage of centrioles (identified by SAS-6 surrounded by SPD-2) with or without a neighboring immature procentriole (identified by SAS-6 not surrounded by SPD-2) during the indicated stages. Nuclei in mitosis were identified by DNA staining as having the most condensed chromatin in the miotic region of the gonad; *N* = Mitotic zone (G1), S, G2-phase: 37, Mitosis (mitotic zone): 7, early meiotic prophase: 46. Data underlying the graphs shown in the figure can be found in S1 Data. SD, standard deviation; SIM, Structured Illumination Microscopy; U-Ex-STED, Ultrastructure Expansion coupled with STimulated Emission Depletion.

We investigated the distribution of 12 centriolar and PCM core components. As detailed in Table 1 and the Materials and methods section, with the exception of mCherry::HYLS-1, we visualized each protein as an endogenously N-terminally [N] or C-terminally [C] tagged component, a tagged version expressed under the endogenous promoter in the absence of the endogenous component and/or previously validated antibodies against the endogenous protein. We found that only 3 of the 12 components, SAS-6, SAS-5, and SAS-4, localize to both centriole and procentriole during S and G2. Using signal intensity in 3D-SIM images as a proxy for protein amount, we found that there is an undistinguishable amount in the centriole and the procentriole for both SAS-5 and SAS-6 (Fig 2A). In contrast, the amount of SAS-4 in the procentriole is on average approximately 10 times lower than it is in the centriole and also exhibits large variability (Fig 2A).

**Table 1:** List of reagents to detect centriolar proteins.

Protein	Reagent 1	Reagent 2	Reagent 3
SAS-6	3xFlag N-terminal CRISPR tag (this study).	Single copy C-terminal GFP tag expressed under the endogenous promoter without the endogenous locus [57].	AB raised in rabbit against full-length protein [43].
SAS-5	N-terminal AB raised in rabbit against aa 1–122 [58].		
SAS-1	3xFlag N-terminal CRISPR tag (this study).	3xFlag C-terminal CRISPR tag (this study).	
SAS-4	AB raised in rabbit against aa 350–517 [19].	GFP C-terminal CRISPR tag [59].	
α-tubulin	N-terminal AB raised in rabbit against the first 100 aa (EP1332Y, Abcam, ab52866).	C-terminal antibody raised in rat against the C-terminal Tyrosine α-tubulin (EMD Millipore, MAB1864).	
HYLS-1	Single copy N-terminal mCherry tagged transgene expressed under the endogenous promoter in presence of endogenous HYLS-1 [60].		
PCMD-1	Single copy N-terminal GFP tag expressed without the endogenous locus [21].	Single copy C-terminal GFP tag expressed without the endogenous locus [21].	
SPD-5	AB raised in rabbit against the C-terminal 18 aa [24].		
SPD-2	GFP C-terminal CRISPR tag [61].	tagRFP N-terminal CRISPR tag [62].	
SAS-7	tagRFP N-terminal CRISPR tag [62].		
TBG-1	AB raised in rabbit against the C-terminal 17 aa [28].		
MZT-1	GFP N-terminal CRISPR tag [27].		

In addition to the invariable presence of SAS-6 and SAS-5 at the procentriole, we found using U-Ex-STED that ZYG-1 accumulates at the base of the procentriole in S and G2 in the mitotic region, as well as during early meiotic prophase (S1B Fig). In contrast to SAS-6 and SAS-5, ZYG-1 levels in the centrioles located in the mitotic zone exhibit high variability. We speculate that levels of ZYG-1 are higher in S phase and lower in G2 in the mitotic region because low levels were also observed during the prolonged G2 of meiotic prophase (S1B Fig). Moreover, we found SPD-2 to be present in a ring around the centriole, abutting the base of the procentriole (Fig 2B). Interestingly, SPD-2 radial distribution is not uniform but often enriched at the site of procentriole formation as evidenced by line scan analysis (Fig 2B and 2C, 22/33 cases). We speculate that such an enrichment may reflect ZYG-1-mediated modification of SPD-2 to serve as a platform for procentriole formation or else local increase of SPD-2 as a result of procentriole formation.

Previous analysis in the one-cell stage embryo established that the procentriole acquires SAS-4 and microtubules after SAS-6/SAS-5 recruitment [7]. Using U-Ex-STED, we found in the gonad that the procentriole likewise harbors little SAS-4 initially and that more protein is recruited at prometaphase, resulting in similar levels of SAS-4 in the centriole and the procentriole by then (Fig 2D). This maturation coincides with the loading of microtubules onto the procentriole (S1C Fig). As expected from these observations, approximately 90% of centrioles harbor an immature procentriole during S and G2 phases in the mitotic region, while only centrioles without an accompanying procentriole are observed by the time of mitosis, when the procentriole disengages and matures into a centriole (Fig 2E). Furthermore, during the prolonged G2 of meiotic prophase that follows, all centrioles are again accompanied by an immature procentriole (Fig 2E). These observations taken together indicate that centriole formation in the gonad is characterized by 2 steps: an initial rapid formation of a procentriole harboring SAS-6 and SAS-5, followed briefly before M phase by the recruitment of other components, including SAS-4 and microtubules. Interestingly, this coincides with the time during which centrioles recruit PCM and start to organize the spindle (see below and reviewed in [20]), potentially suggestive of a functional link between procentriole maturation and PCM expansion.

### U-Ex-STED reveals consecutive ring-like distribution of *C*. *elegans* centriolar proteins

**Fig 3:**
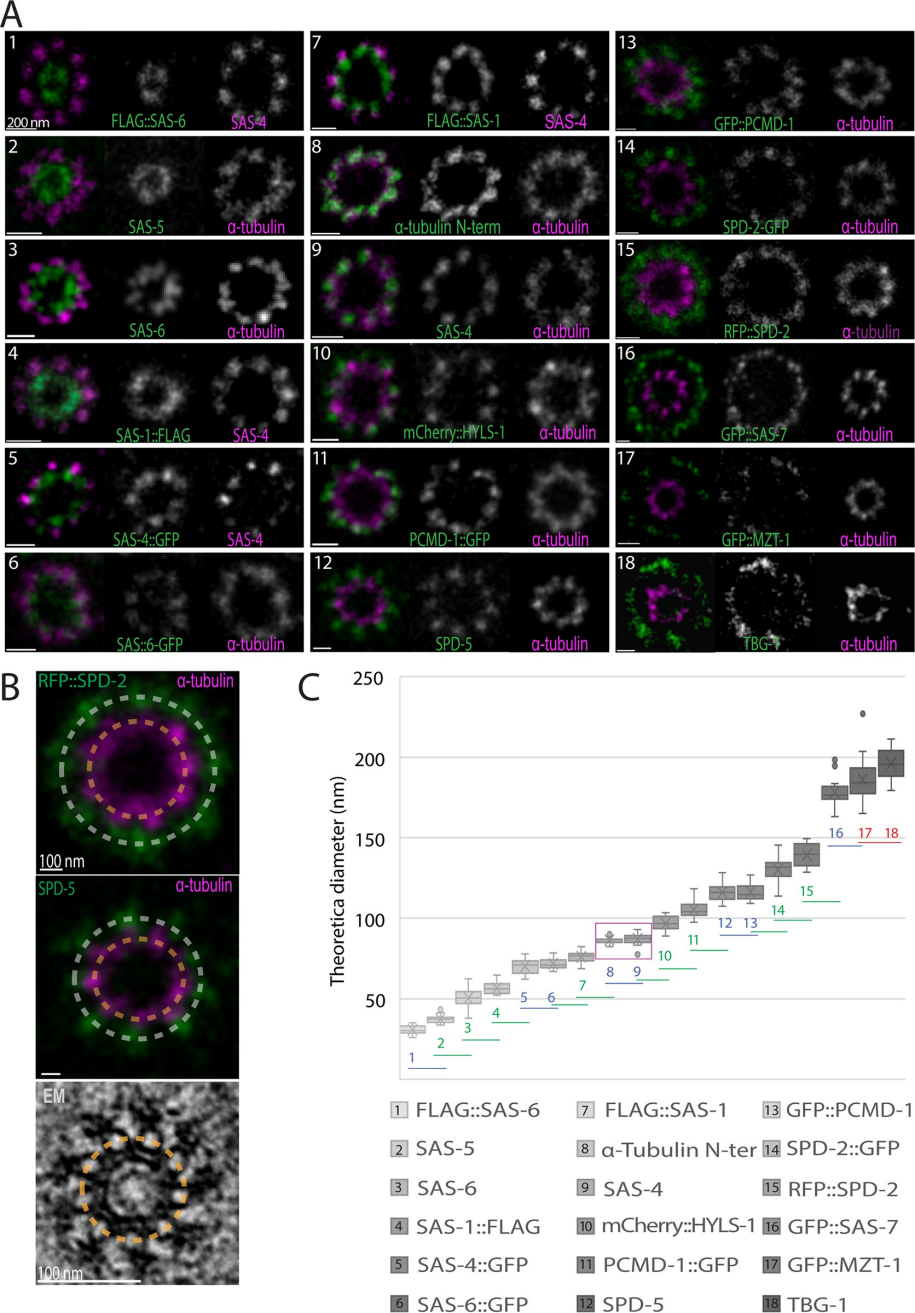
Relative position of centriolar components within the centriole. (A) U-Ex-STED of centrioles from early meiotic prophase stained for the indicated proteins. Each component (green) was imaged together with either α-tubulin (visualized with an antibody recognizing the C-terminus of the protein) or SAS-4 (visualized with an antibody raised against amino acid 350–517 of the protein) (both magenta), which served as standards for the quantification in C. (B) Examples of fitted rings on fluorescent signal used to calculate the diameter of each component relative to α-tubulin or SAS-4 standards. In each image, the diameter of the centriolar component (in these cases RFP::SPD-2 (top) and SPD-5 (middle)) and that of the α-tubulin signal were measured along the dashed lines. The perimeter of the centriolar component was then divided by that of the α-tubulin signal. To obtain the theoretical diameter of the component before expansion, this value was normalized by the diameter of microtubules in EM images of centrioles (see Fig 5). (C) Calculated diameter of each centriolar component as determined in (B), arranged from the smallest to the largest. Magenta box highlights α-tubulin (#8) and SAS-4 (#9). Numbers in the graph indicate the identity of the component. Colors indicate whether the diameter is significantly different from 0 (red), 1 (blue), or 2 (green) neighboring values (Student two-tailed *t* test, significance *p* < 0.005); the lines below the numbers indicate pair-wise comparisons with the neighbor on the right. The middle line of the boxplots corresponds to the median, the cross represents the mean, the box includes 50% of values (IQR), and the whiskers show the range of values within 1.5*IQR. *N* = Flag::SAS-6: 21, SAS-5: 25, SAS-6: 22, SAS-1::FLAG: 10, SAS-4::GFP: 21, SAS-6::GFP: 15, FLAG::SAS-1 15, α-tubulin (N-ter): 19, SAS-4: 22, mCherrry::HYLS-1: 20, PCDM-1::GFP: 24, SPD-5: 20, GFP::PCDM-1: 21, SPD-2::GFP: 25, RFP::SPD-2: 20, GFP::SAS-7: 15, GFP::MZT-1: 20 and TBG-1: 20. Data underlying the graphs shown in the figure can be found in S1 Data. EM, electron microscopy; U-Ex-STED, Ultrastructure Expansion coupled with STimulated Emission Depletion.

We proceeded to comprehensively uncover the precise distribution of centriolar and PCM core proteins using U-Ex-STED. We used top views of centrioles to determine the radial distribution of these components (Fig 3A). Remarkably, except for ZYG-1 (see S1B Fig), such top views revealed that all components exhibit a ring-like distribution, with distinct diameters. To analyze the position of each component with respect to the others, we set out determine the diameter of each ring relative to that of microtubules, which were used as an invariant reference in this analysis (Fig 3B). To verify the validity of this approach, we costained α-tubulin with 2 different antibodies, finding that the 2 signals colocalize and that the corresponding rings hence exhibit the same diameter (without correction for the expansion factor: C-terminus 458 ± 38 nm, N-terminus 455 ± 35 nm, *p* = 0.81, *N* = 19; Fig 3A). Furthermore, an antibody raised against the middle portion of SAS-4 likewise had the same perimeter as α-tubulin, in line with the fact that the SAS-4-relative CPAP is a microtubule binding protein (Fig 3A and 3C, #9) [40–42]. Thus, α-tubulin and SAS-4 can be used interchangeably as invariant references in this analysis.

To estimate the ring diameter of each component in nonexpanded samples, we determined the diameter of the ring formed by the 9 microtubules in a novel EM data set of early meiotic prophase centrioles to be 87.9 ± 5.7 nm (*N* = 44; see below) and compared this value to the α-tubulin signal diameter determined with U-Ex-STED (Fig 3B). Moreover, we found that the α-tubulin diameter determined with U-Ex-STED following correction of the expansion factor (5.2) is similar to that measured for microtubules by EM (88 ± 8 nm; *N* = 38). This standardization method enabled us to estimate the actual diameter of the ring distribution of each protein, going from the smallest one, SAS-6[N], to the largest ones, SAS-7[N], MZT-1, and TBG-1 (Fig 3C). This analysis established that most components that were shown previously through biochemical and cell biological assays to physically interact are indeed located in close vicinity to one another. This is the case for SAS-6 and SAS-5 [43], SAS-4 and HYLS-1 [17], SAS-7 and SPD-2 [8,44], SPD-2 and SPD-5 [45], as well as PCMD-1 and SAS-4 or SPD-5 [46].

Overall, U-Ex-STED enabled us to localize in a comprehensive manner centriolar and PCM core component with unprecedented spatial precision.

### 9-fold symmetrical distributions and chiral elements in *C*. *elegans* centriole

**Fig 4:**
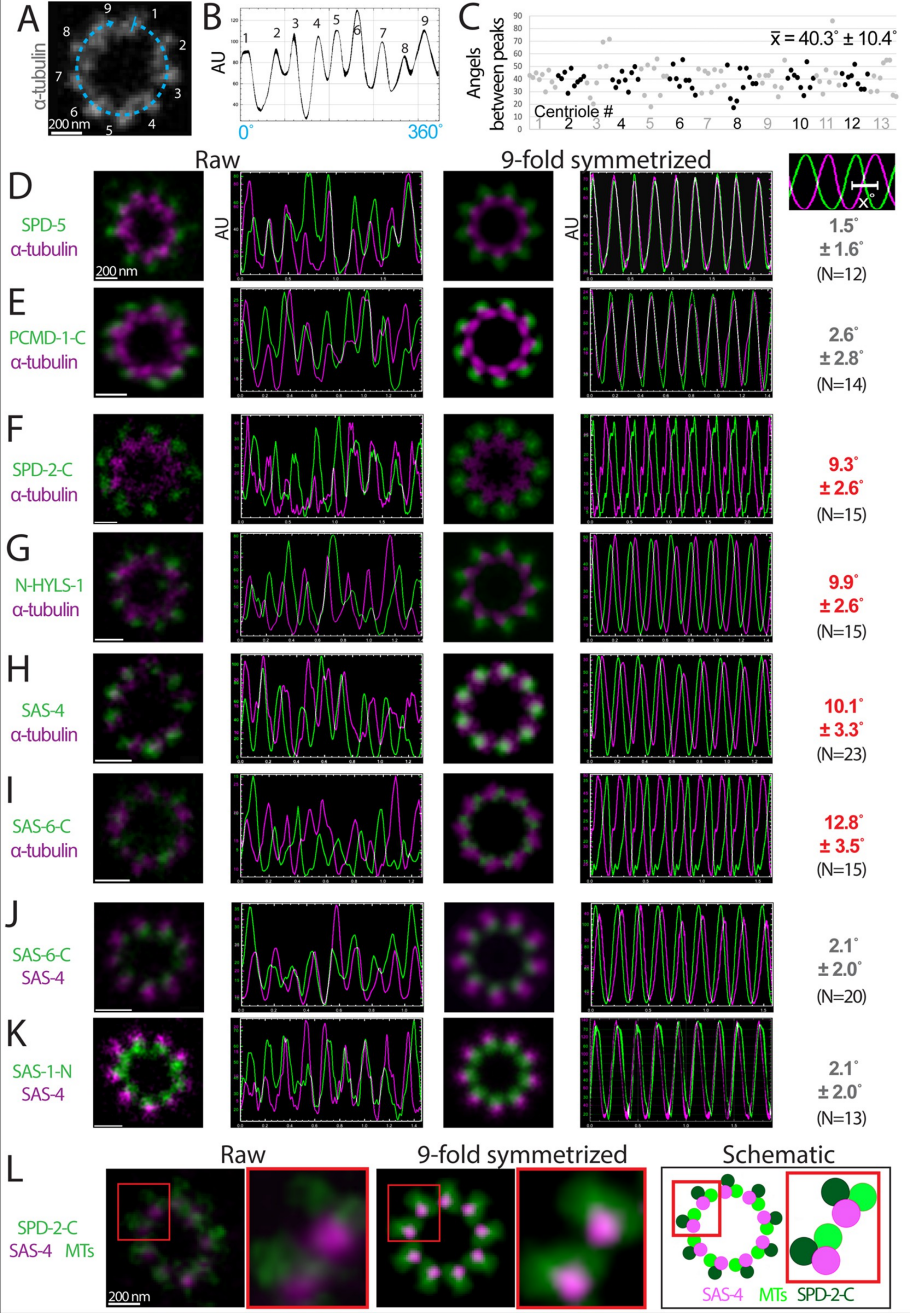
Chiral features of the centriole. (A) U-Ex-STED of a centriole stained for α-tubulin. Numbers correspond to the signal peaks in the intensity profile along the dashed line reported in (B). (B) Signal intensity profile along the dashed line in (A) (4 pixels wide). (C) Angles between peaks of α-tubulin signal intensity profiles in 14 top views of centrioles imaged with U-Ex-STED. Angles were determined by dividing the distance between each neighboring peak by the length of the entire profile, multiplied by 360. Alternating grey and black data points indicate values from each of the 14 centrioles examined. 11/14 centrioles showed 9 clearly discernable signal peaks, 2/14 only 8, and 1/14 apparently 10 such peaks. (D-K) U-Ex-STED and plot signal intensity profiles of raw images (left two panels) and corresponding 9-fold symmetrized versions (right) of the indicated pairs of components (green and magenta). In cases top views were slightly tilted, images were circularized before 9-fold symmetrization using the Fiji plugin “Transform-Interactive Affine”. The numbers on the very right represent the average offset of the 2 nearest signal intensity peaks in 9-fold symmetrized images for the 2 components tested. (L) (Left) U-Ex-STED of centriole from a worm expressing SPD-2::GFP stained for GFP and α-tubulin in the same color (green), as well as for SAS-4 (magenta). (Middle) Corresponding 9-fold symmetrized version. (Right) Schematic representation of the IF analysis, manually separating the SPD-2::GFP and α-tubulin signals based on their ring diameter. Red boxes are magnified on the right of each image. Note that we have not analyzed HYLS-1 in this manner, as the mCherry::HYLS-1 signal intensity was too weak to this end. Data underlying the graphs shown in the figure can be found in S1 Data. IF, immunofluorescence; U-Ex-STED, Ultrastructure Expansion coupled with STimulated Emission Depletion.

We next addressed whether the ring-like distribution of each centriolar and PCM core component exhibits 9-fold radial symmetry. To this end, we conducted an analysis of the U-Ex-STED data set for each component that is illustrated in the case of α-tubulin in Fig 4A and 4B. First, a circle was drawn along the ring-like signal and an intensity profile measured along this circular line (Fig 4A and 4B). In the majority of cases, this yielded 9 clearly distinguishable peaks. In ideal top views, with no or very little tilt of the organelle with respect to the imaging axis, the average distance between signal peaks is consistent with the 40° angle expected from a 9-fold radially symmetric structure (Fig 4C). Importantly, besides α-tubulin, we found a 9-fold symmetric arrangement for SPD-5, PCDM-1[C], SPD-2[C], HYLS-1[N], SAS-4, SAS-6[C], and SAS-1[N] (Fig 4D–4K, left two panels, raw).

We next investigated whether centriolar proteins thus localized exhibit an offset distribution with respect to microtubules. To that end, we examined if the 9-fold radial symmetric distributions are on the same angular axis as the microtubules using the following analysis pipeline. First, we averaged the signals of the microtubules and of the component to be tested by applying 9-fold symmetrization (S2 Fig; [47,48]). Second, we acquired a signal intensity plot along the ring in the resulting symmetrized images for both channels. As expected, given the 9-fold symmetrization, in such an analysis individual signal peaks for each channel are approximately 40° apart (360°/9 signal peaks) (Fig 4D–4K). Third, signal intensity plots from the 2 channels are overlaid, and the angular distance between each peak in the α-tubulin channel and the neighboring peak in the second channel determined. In this manner, the average angular offset in each centriole of the component in question versus microtubules is computed.

Strikingly, the above analysis pipeline revealed that components exhibiting a 9-fold symmetric arrangement fall into 2 well-separated groups. In a first two-membered group containing SPD-5 and PCMD-1[C], the offset with respect to microtubules is marginal (<3°) (Fig 4D and 4E), similar to that of the 2 antibodies raised against α-tubulin (2.4° ± 1.6, *N* = 10). Therefore, SPD-5 and PCMD-1[C] are not offset with respect to the microtubules. In stark contrast, a second group of components exhibited a clear offset (>9°) with respect to the microtubules, thus leading to a chiral ensemble (Fig 4F–4K). This second group includes SPD-2[C] and HYLS-1[N], which are both located outside the microtubules (Fig 4F and 4G). In addition, SAS-4 and SAS-1[N], which both have a ring diameter similar to that of α-tubulin, exhibited an offset with respect to microtubules (Figs 3C and 4H). More internally, SAS-6[C] also exhibits a strong offset with respect to microtubules (Fig 4I). Importantly, we found additionally that SAS-4 and SAS-6[C] are well aligned with one another (Fig 4J), as are SAS-4 and SAS-1[N] (Fig 4K). Thus, the offset of SAS-4 exhibits the same handedness with respect to microtubules as that of SAS-6 and SAS-1.

How does the offset handedness of components located outside the microtubules relate to that of those located more centrally? To address this question, we set out to simultaneously examine offset in the angular axis with respect to microtubules of the outer components SPD-2[C] and the inner offset component SAS-4. Because our microscopy setup does not lend itself to performing high-quality 3-color STED, we marked SPD-2[C] and α-tubulin in the same color in this experiment, since they exhibit clearly distinct ring diameters (see Fig 3A and 3C, #8 and #14). Importantly, this analysis uncovered that SPD-2[C] and SAS-4 are invariably located on the same side of the microtubules (Fig 4L). Note that this is regardless of whether SPD-2[C] and SAS-4 are to the left or to the right of the α-tubulin signal, which is expected to depend on whether a centriole is viewed from the proximal or the distal end. We conclude that the offset of the outer component SPD-2[C] and the inner component SAS-4 has the same handedness with respect to microtubules.

### Ultrastructural map of the *C*. *elegans* gonad centriole

**Fig 5:**
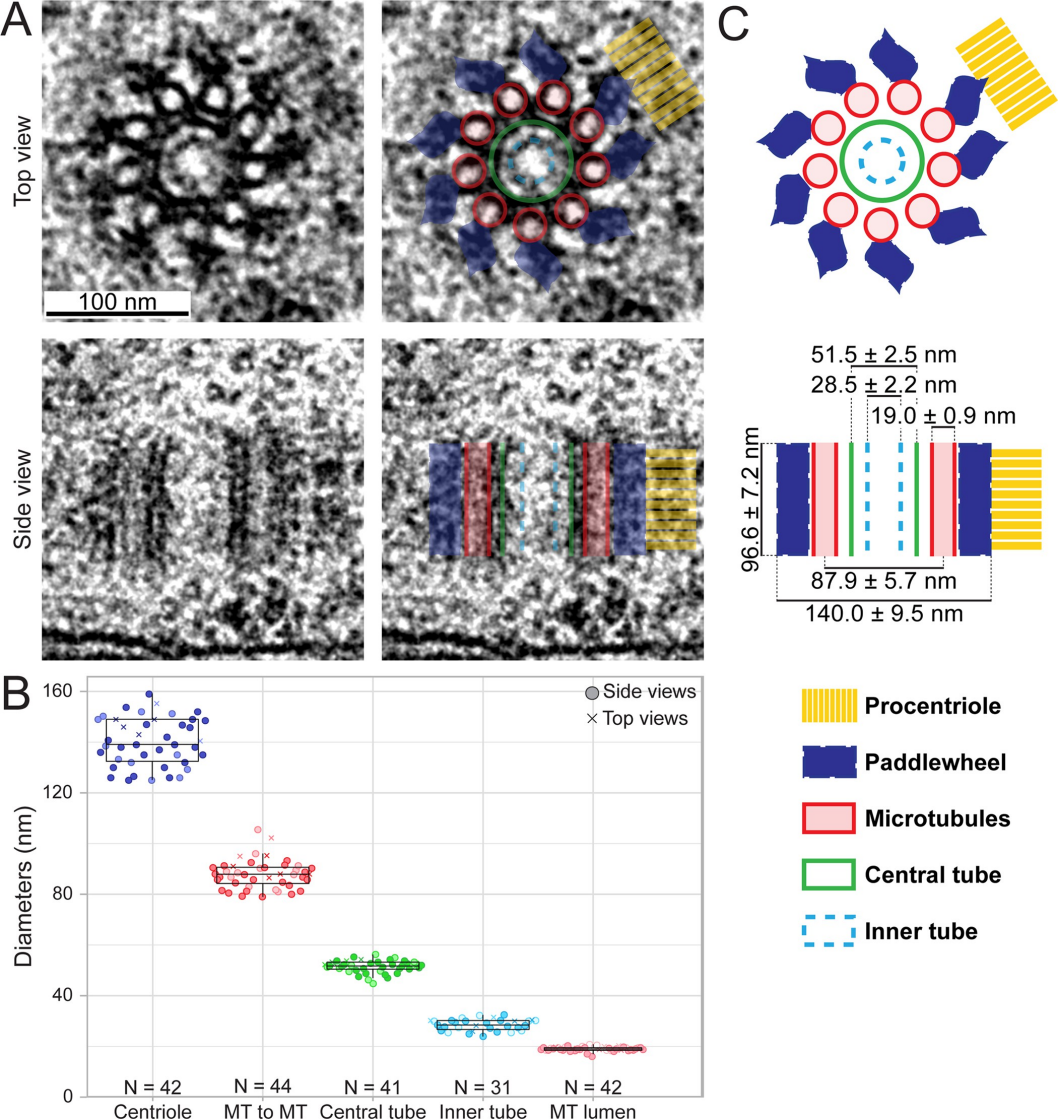
EM analysis of the centriole in the gonad. (A) (Left) EM of top and side views, as indicated, of early meiotic prophase centrioles. (Right) Overlay with distinct ultrastructural compartments as described in (C). (B) Diameters of ultrastructural compartments of the gonad centriole. Top view (crosses) and side views (circles) were analyzed; light and dark shade of colors represent data points from 2 independent samples. The middle line of the boxplots corresponds to the median, the box includes 50% of values (IQR), and the whiskers show the range of values within 1.5*IQR. (C) Schematic representation of top and side views of centrioles with ultrastructural compartments depicted in colors, as well as average measurements ± SD (see also C). *N* = 38 for centriole height. Data underlying the graphs shown in the figure can be found in S1 Data. EM, electron microscopy; SD, standard deviation.

Having achieved precise protein localizations in the gonad centriole with U-Ex-STED, we set out to determine whether we can assign specific centriolar components to specific compartments of the organelle. Given that the ultrastructure of the worm centriole has been best studied in the early embryo [6–8], and considering that the centriole may exhibit tissue-specific features, we set out to characterize the ultrastructure of the gonad centriole. Using Correlative Light Electron Microscopy (CLEM) of chemically fixed samples to ease spotting of G2 early meiotic prophase centrioles followed by 50 nm serial sectioning, we acquired the largest EM data set of worm centrioles to date (*N* = 44). As shown in Fig 5A and 5B, as well as in S3A Fig, we found that peripheral paddlewheels, microtubules, central tube, and inner tube are all clearly discernable, as they are in the early embryo [6–8]. However, side views established that the centriole is shorter in the gonad than in the early embryo (96.6 ± 7.2 nm as compared to approximately 175 nm [7,8]). Moreover, top view revealed that the paddlewheels are slightly smaller as well [8]. Apart from these 2 differences, we conclude that the overall ultrastructure of the centriole is conserved between the embryo and the gonad.

Using both top and side views, we determined the diameter of the entire organelle, encompassing the most peripheral paddlewheel features, to be 140.0 ± 9.5 nm (Fig 5B and 5C). Moreover, the diameters of central tube and inner tube are 51.5 ± 2.5 nm and 28.6 ± 2.3 nm, respectively, whereas that of the radial array of the microtubules is 87.9 ± 5.7 nm (Fig 5C). These measurements are in line with those from high-pressure frozen early embryonic centrioles [7,8], indicating that there is no or marginal shrinkage due to chemical fixation.

The paddlewheels of the centriole in the embryo were reported to exhibit a clockwise twist with respect to the microtubules when viewed from the distal end, using the presence of the procentriole to define the proximal end of the centriole [8]. In the gonad, however, where the centriole is shorter, the procentriole often appears to cover the centriole height in its entirety (S3B Fig; *N* = 15). In those cases where the centriole is higher than the cross-sectional diameter of the procentriole (*N* = 17), the latter could sometimes emanate from the vicinity of one of the 2 ends (S3C Fig). The observations made by EM were supported and complemented by analyses conducted with U-Ex STED. Thus, we found that when the centriole is short, the procentriole emanates from its entire height (S3D Fig, left). When the centriole is higher, the procentriole can be either centered with respect to the centriole (S3D Fig, middle) or positioned closer to one end (S3D Fig, right). Therefore, we speculate that, at the least in the gonad, the procentriole can emanate from any site along the pourtour of the centriole. Regardless, chirality of the centriole cannot be assessed reliably with respect to procentriole orientation in the gonad. Nevertheless, the fact that the procentriole can also emanate from the middle of the centriole raises the possibility that centriole chirality might not be fixed with respect to procentriole orientation.

### Establishing the molecular architecture of the *C*. *elegans* centriole: Beyond microtubules

**Fig 6:**
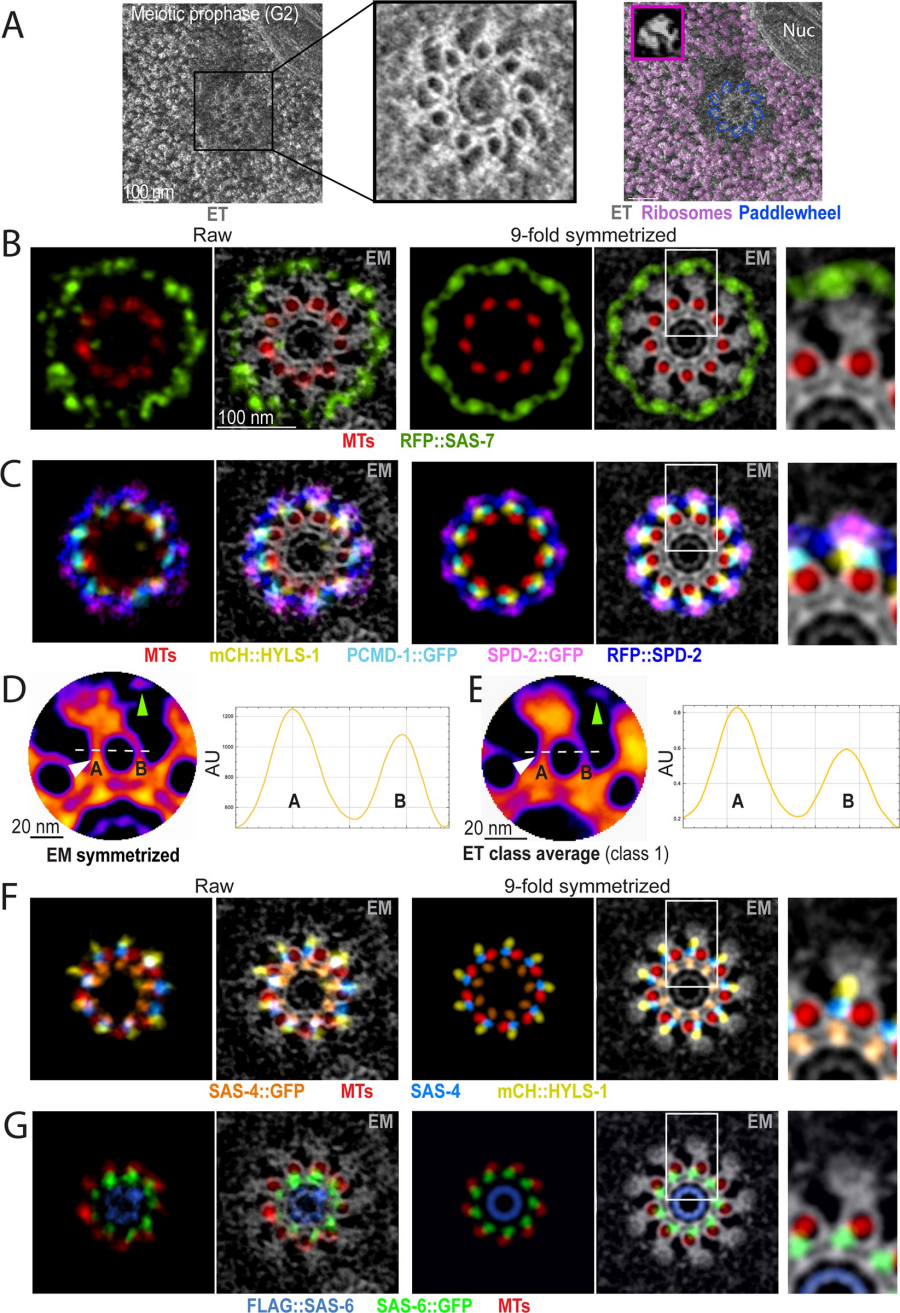
Overlay of EM and U-Ex images. (A) (Left) Max intensity Z-projection of ET of an early meiotic prophase centriole and surrounding region. (Middle) Magnification of the black box in the image on the left. (Right) Manually annotated ribosomes are shown in magenta and paddlewheel structures with dark-yellow outlines. Note that the ribosome-free area extends beyond the paddlewheels. Purple inset shows a magnified ribosome from the same ET image. (B, C, F, G) Overlay of U-Ex-STED and EM images (inverted grey levels) of centrioles from early meiotic prophase. Circularized images (left two panels), corresponding 9-fold symmetrized versions (next two panels), and magnification of the insets highlighted by the white box (very right). (B) Note that SAS-7 extends beyond the paddlewheel. (C) Overlay of paddlewheel components. (F) Overlay of components around microtubules. (G) Overlay of SAS-6 (N- and C-ter) and microtubules. (D, E) (Left) Magnification of a 9-fold symmetrized centriole imaged by EM (D) and highest populated class from class-averaging of particles containing microtubules and paddlewheels from individual ET tilt series of four centrioles (E) (see S4 Fig). Images are colorized with the LUT “Fire” (low intensities in blue, high intensities in magenta and red). Light green arrowheads point to the small density next to the paddlewheel (IPD), filled white arrowheads to that spanning from the central tube to 1 side of the microtubule (SCD). (Right) Intensity profiles were obtained along the indicated dashed lines (10 pixels wide). Microtubules display consistently more density on the side located under the paddlewheel (marked with an A) compared to the other side (marked with a B). EM, electron microscopy; ET, electron tomography; IPD, Inter Paddlewheel Density; SCD, SAS-6/4/1 Containing Density; U-Ex-STED, Ultrastructure Expansion coupled with STimulated Emission Depletion.

To better understand the cellular context in which the centriole resides, we conducted tomographic analysis of the EM sections (ET), which revealed a ribosome free area approximately 262 ± 26 nm in total diameter extending beyond the paddlewheels (Fig 6A; *N* = 3). This diameter is approximately 60 nm larger than that of the largest ring-like distribution observed in this work (see Fig 3C), raising the possibility that other proteins may be present in this area.

We set out to determine the identity of the centriolar and PCM core proteins that correspond to given ultrastructural compartment of the organelle. To this end, we devised a method that relies on overlaying U-Ex-STED and EM images, using microtubules as a joint registration standard. In brief, we circularized, rotated, and size-adjusted jointly the 2 U-Ex-STED channel signals, aligning the α-tubulin signal with the microtubules in the EM images (S4 Fig). We applied this method initially on the symmetrized images and then likewise adjusted the raw data (S4 Fig). We report the results of this analysis hereafter, starting with the outside of the organelle.

Overlaying the U-Ex-STED and EM data revealed that SAS-7[N] localizes just outside the paddlewheel, partially filling the region devoid of ribosomes surrounding the centriole (Fig 6B). Four components were found to localize to the paddlewheel: HYLS-1[N], SPD-2, SPD-5, and PCMD-1. SPD-5 and PCMD-1[C] are on the same angular axis as microtubules in the U-Ex-STED data set (see Fig 4D and 4E), and we indeed find PCMD-1[C] just outwards of microtubules in the overlay, constituting the base of the paddlewheel (Fig 6C). HYLS-1[N] also localizes to the base of the paddlewheel, but in contrast to SPD-5 and PCMD-1[C], it does so with an offset with respect to the microtubules (Fig 6C). SPD-2 is the outermost component of the paddlewheel with the 2 ends showing distinct distributions: SPD-2[C] appears as foci positioned just outside of microtubules, with an angular offset with respect to them (Fig 6C; see also Fig 4F), whereas SPD-2[N] localizes slightly further to the outside as an epitrochoid with 9 lobes extending left and right over the paddlewheel (Fig 6C). Interestingly, we detected a previously unnoticed small electron-dense region in the EM and ET data sets (see below) located between neighboring paddlewheels (Fig 6D and 6E, green arrowheads), which can be partially matched with the position of SPD-2[N] in these overlays. We name this density Inter Paddlewheel Density (IPD). Overall, this analysis reveals in exquisite detail the molecular architecture of components located outside the centriolar microtubules.

### Molecular architecture at the level of the microtubules

We next report the analysis of components located more centrally. Upon careful analysis of the symmetrized EM data set, we noticed another novel density, which starts from the central tube (Fig 6D, dashed arrowhead), extends towards and along each microtubule, rendering 1 side of the microtubule more pronounced than the other (Fig 6D, white arrowhead). This density displays the same angular offset with respect to the microtubules as the paddlewheel. Since microtubules are not always perfectly perpendicular to the plane of sectioning, we performed ET to obtain bona fide top views of microtubules and thus better analyze this novel density. From individual tilt series of 4 centrioles, we picked 628 particles containing microtubules and paddlewheels; class-averaging resulted in 3 well-defined classes containing 92% of input particles (S5A Fig). In all 3 classes, the novel density is present on the side of the microtubule above which the paddlewheel is located (Figs 6E and S5B). Given that SAS-6, SAS-4, and SAS-1 all display the same angular offset direction with respect to microtubules as the paddlewheel component SPD-2[C] (see Fig 4F and 4H–4L), we propose that these 3 proteins together could compose this novel offset density. Therefore, we name this novel density “SAS-6/4/1 Containing Density” (SCD). Overlays of the corresponding U-Ex-STED and EM images indeed revealed perfect alignment of SAS-4, SAS-6[C], and SAS-1[N] with the SCD, below 1 side of the microtubule (Figs 6F, 6G, and S5C). Moreover, SAS-4[C] overlaps almost perfectly with the SCD at the level of the central tube, whereas SAS-1[N] has an indistinguishable diameter from SAS-6[C] (see Fig 3C). Taken together, our data suggest that SAS-4, SAS-6, and SAS-1 form the newly described chiral SCD, with SAS-4 potentially bridging it to HYLS-1.

### The N-terminus of SAS-6 is present at the inner tube and does not form a spiral

We capitalized on the unprecedented high resolution afforded by U-Ex-STED to address whether *C*. *elegans* SAS-6 forms a ring or instead a steep spiral in vivo, as has been hypothesized based on structural and biophysical data [16]. The spiral model predicts that SAS-6[N] should be apparent in top views as a small ring with a diameter of approximately 4.5 nm [16]. Given the approximately 14 nm effective lateral resolution achieved using U-Ex-STED, this would appear as a single focus. Contrary to this prediction, we found that the diameter of the ring formed by SAS-6[N] is approximately 31 ± 3 nm, overlapping with the inner tube in EM images (Fig 6G). We noted also that SAS-6[C] localizes approximately 41 ± 4 nm away from SAS-6[N], in line with the fact that the coiled-coil domain of SAS-6 is approximately 35 nm long and followed by an intrinsically disordered region of approximately 90 amino acids [49]. Taken together, our observations indicate that, rather than a steep spiral, in vivo, *C*. *elegans* SAS-6 forms a ring-containing cartwheel.

## Discussion

**Fig 7:**
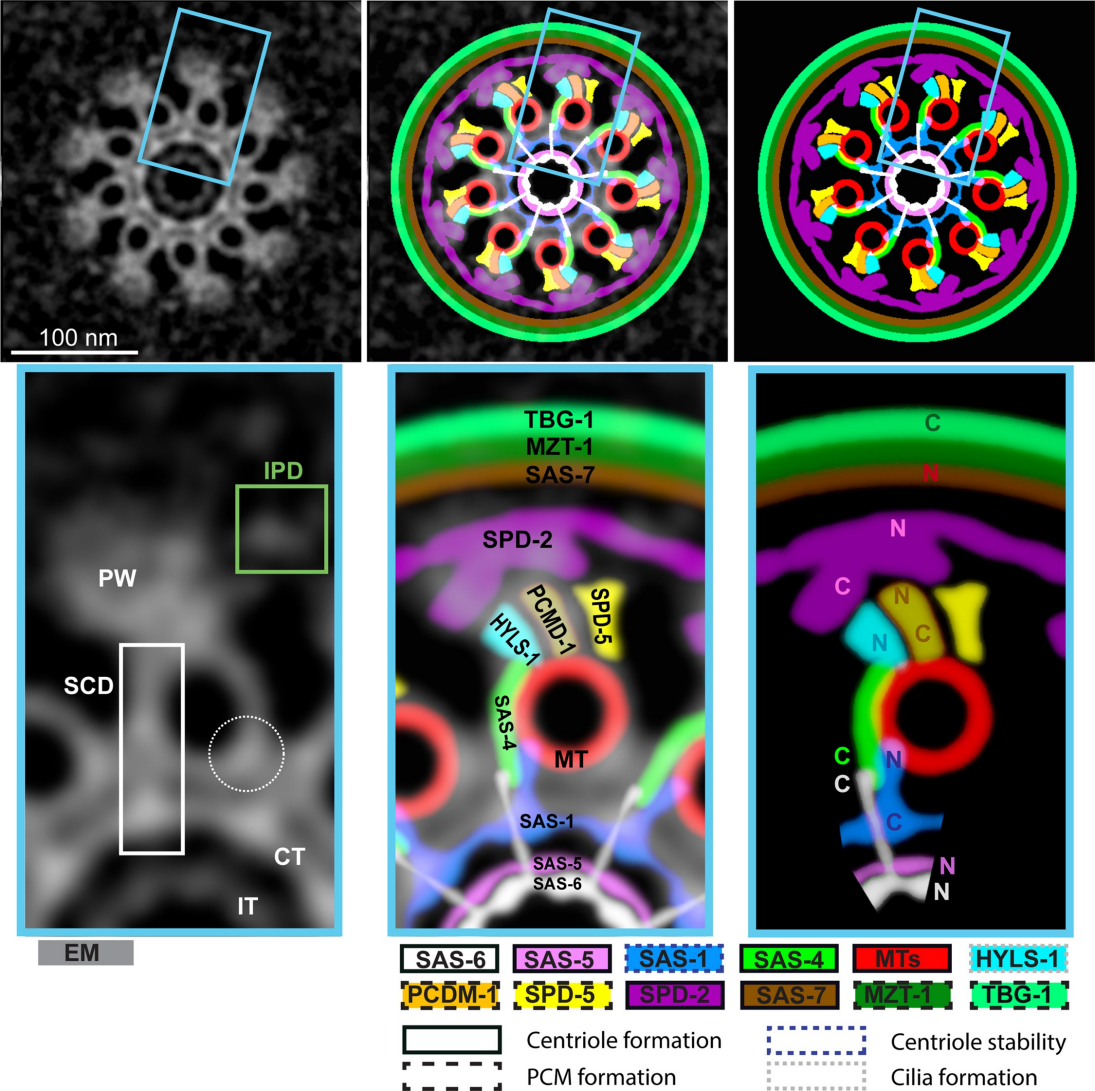
Schematic representation of the localization of components within the centriole. (Top left) 9-fold symmetrized EM image of a centriole. (Top middle) Overlay of the image on the left with corresponding schematic representation on the right. (Top right) Coarse grained schematic representation of the localization within the centriole of components analyzed in this study. (Bottom left) Magnification of the blue boxed region from the image above. Green box highlights the IPD, white box the SCD. Dashed circle highlights a small density to which no known centriolar protein has been assigned (see Discussion). (Bottom middle and right) Magnifications of the blue boxed regions from the images above, with an indication of the localization of each protein, including where the N- and C-terminus maps when known. No mention of termini is indicated when merely antibodies were utilized. EM, electron microscopy; IPD, Inter Paddlewheel Density; SCD, SAS-6/4/1 Containing Density.

We deciphered the molecular architecture of the minute *C*. *elegans* centriole in unprecedented detail by combining U-Ex-STED with EM, thereby localizing 12 centriolar and PCM core proteins to distinct ultrastructural compartments (Fig 7).

The precise localization of proteins achieved herein is by and large compatible with, and extends, previous findings. Thus, components that were shown previously through biochemical and cell biological assays to physically interact are indeed located in close vicinity of one another in our map, including SAS-6 and SAS-5 [43], SAS-4 and HYLS-1 [17], SAS-7 and SPD-2 [8,44], SPD-2 and SPD-5 [45], as well as PCMD-1 with SAS-4 and SPD-5 [46]. Other distributions were not necessarily anticipated from prior work. For instance, we found that SAS-7 localizes partly outside the paddlewheel structure, within the zone of ribosome exclusion. SPD-2 and PCMD-1 are not needed for SAS-7 localization, whereas SAS-7 is needed for normal centriolar levels of SPD-2 and PCMD-1, as well as for integrity of the paddlewheels themselves [8,46]. The localization of SAS-7 outside of SPD-2 and PCMD-1 raises the possibility that SAS-7 functions through a shielding mechanism rather than by recruiting SPD-2 and PCMD-1. Another functionally suggestive distribution uncovered here is that of SAS-1: SAS-1[N] localizes just inside the microtubule ring, in line with the fact that this part of the protein associates with and stabilizes microtubules when ectopically expressed in human cells [18]. Therefore, it is tempting to speculate that *C*. *elegans* SAS-1 maintains centriole integrity by locally exerting a microtubule stabilizing function. The only reported interaction potentially not recapitulated here is that of microtubules with SAS-5, which is mediated by a N-terminal region encompassing amino acids 90-265 and result in colocalization with the microtubule network upon transfection in COS-7 cells [50]. We used antibodies raised against amino acids 1-122 of SAS-5 and found them to localize approximately 50 nm more centrally than the centriolar microtubules, raising the possibility that the SAS-5 N-terminus interacts preferentially with another component in the *C. elegans* centriole.

Overlaying EM images with U-Ex-STED images of all known centriolar proteins to date allows us to consider whether there may be centriolar proteins that have not yet been identified in the worm. Such proteins might correspond to EM densities that cannot be readily accounted by the distribution of the proteins assessed here by U-Ex-STED. One such density, distinct from the ICP and the SCD, is apparent on the inner side of microtubules, opposite the SCD (see Fig 7, dashed circle). This is reminiscent of the region to which Cep135 and Cep97 localize in the fly centriole [34]. It has been suggested that a divergent Cep135 protein localizes to centrioles of *C*. *elegans* during certain developmental stages [51], and it will be interesting to use U-Ex-STED to address whether it maps to this density. Alternatively, it is possible that a segment of the proteins tested here and localizing to such a density would not be apparent from tagging the N- or C-terminal part of the protein, or with some of the antibodies that were utilized.

Our approach enabled us to probe the higher order oligomerization mechanisms of SAS-6 in vivo. Previous structural and biophysical experiments led to the suggestion that such oligomers form a steep spiral instead of a ring as in other systems [16]. The steep spiral model predicts that SAS-6[N] appears in top views as a small ring with a diameter of approximately 4.5 nm [16]. We found instead that SAS-6[N] forms a ring approximately 31 ± 3 nm in diameter, which neatly overlays with the inner tube ultrastructural compartment in EM images. We conclude that SAS-6 does not form a steep spiral in the worm and propose instead that the protein assembles into a ring-containing cartwheel as in other organisms. Alternatively, *C*. *elegans* SAS-6 may assemble into a shallow spiral. Moreover, we found SAS-6[C] to be positioned approximately 41 ± 4 nm away from SAS-6[N], compatible with a cartwheel structure in which the SAS-6 coil-coil domains form spokes extending towards the peripheral microtubules, as in other systems [14,15]. It will be interesting to uncover how the intrinsic properties of *C*. *elegans* SAS-6 that enable it to form a steep spiral in vitro are modulated in the organismal context to adopt a ring-like configuration. This might be aided by interacting proteins, such as SAS-5 [44,50,52], or by a connection of SAS-6[C] to microtubules. Alternatively, properties of the centriole surface from which the procentriole assembles might impose a different conformation, since the presence of a surface can help constrain the inherent helical properties of *Chlamydomonas reinhardtii* SAS-6 polymers into a ring [53].

Our analysis uncovered offset protein distributions with respect to microtubules, thereby resulting in a chiral centriolar ensemble. Such an offset pertains notably to SAS-6[C] and SAS-4, which coincide with the newly identified SCD, an EM density found centrally and laterally to the microtubule wall. Interestingly, the SCD displays an angular offset with respect to the microtubule in the same direction as the paddlewheel and its constituent SPD-2[C]. Angular offsets with respect to centriolar microtubules occur in other systems. For example, EM studies of centrioles in *Trichonympha* and *Chlamydomonas* uncovered that the pinhead component connecting cartwheel and microtubules exhibits an angular offset with respect to the A microtubule that is on the side of the B and C microtubules [54]. Moreover, super-resolution microscopy in *Drosophila* revealed that the centriolar proteins Cep135 and Ana1 exhibit an angular offset with respect to the A microtubule on the side opposite the B microtubule [34].

Chirality of the centriole is a signature feature of the organelle observed across the eukaryotic domain of life. However, the potential evolutionary pressure leading to conservation of such chirality is not clear, although an appealing possibility is that this could be optimal for ciliary and flagellar motility. Regardless, it has been suggested that centriolar chirality may be imparted by inherent chiral features of SAS-6 proteins, with chirality in the inner part of the organelle dictating that of more peripheral elements, including microtubules [53]. Alternatively, chirality could stem from the fact that microtubules of the procentriole grow with a fixed orientation from the surface of the centriole, with the plus end leading. Therefore, the surfaces for molecular interaction available on the left and the right side of a microtubule are inherently different. As a result, a protein that interacts with a specific surface on the microtubule wall and that has a fixed orientation along the polymer, such as SAS-4 (see Fig 7), would necessarily render the centriole chiral. Regardless, it will be important to uncover how chirality of the centriole is established and what its role might be in centriolar biogenesis and function.

It will be of interest to apply the methods developed herein to probe potential variations in the molecular architecture of centrioles in distinct developmental contexts in *C*. *elegans*. For example, we uncovered here that centriole length in the gonad and the early embryo differ substantially; such a height difference may be accompanied by alterations in molecular architecture. Moreover, these methods can be deployed to interrogate with utmost precision the molecular architecture of centrioles in mutant worms in this genetically tractable organism to further unravel mechanisms of organelle biogenesis and function. Beyond *C*. *elegans*, such an analytical framework is anticipated to likewise reveal the distribution of centriolar proteins in other systems, and thereby identify conserved and variable features of organelle architecture.

## Materials and methods

### *C*. *elegans* culture conditions

Worms were grown on *E*. *coli* (OP50) seeded NG agar plates at 20°C and age matched as L1 larvae by bleaching gravid adults according to [63]. Worms were harvested for ethanol fixation or gonad spreading 24 to 36 hours post-L4 stage by washing them off the plate with PBS-T (PBS supplied with 0.1% Tween-20).

### CRISPR/Cas9 genome editing

3xFLAG tagging of SAS-1 and SAS-6 was performed by CRISPR/Cas9 as described in [64]. crRNAs were designed using the GUIDE DESIGN tool (http://crispr.mit.edu). Briefly, young adult worms were injected with CRISPR/Cas9 ribonuclear complexes (homemade, 2.5 μg/μl), and *dpy-10* was used as the coinjection marker. ssDNA repair templates were injected at concentrations of 414 nM for *dpy-10* and 500 nM for the 3xFlag tags, respectively. F1 progenies with roller or dumpy phenotypes were selected and the edits assessed using PCR in the F2 generation, followed by verification with Sanger sequencing.

The crRNA sequences were as follows:

SAS-1 N-terminus: ACAATTACTGGTGCCCTTCT(CGG) (30 μM)

SAS-1 C-terminus: CGGATTTGGAGAATATGATG(AGG) (30 μM)

SAS-6 N-terminus: AATTTTGCTAGTCATTTTTG(TGG) (30 μM)

### Ethanol fixation

Worms were washed twice in PBS-T and kept for 30 to 60 minutes in PBS-T in a 1.5-mL tube to allow emptying of intestines. PBS-T was removed, and 1.5 ml of 100% ethanol then added. Worms were precipitated by gravity, and ethanol then removed completely before resuspension of worms in 25 μL MVD (50% M9, 50% Vectashield (Vector), 7 μg/ml Hoechst (bisBenzimide H 33258)). Fixed worms were pipetted onto a slide, and a 20 × 40 mm ethanol-washed coverslip was applied with slight pressure.

### Gonad spreading

Spreading of *C*. *elegans* gonads was performed in a similar manner as in [39]. Gonads of approximately 1,000 adult worms were dissected in 30 μL dissection solution (0.2 × PBS, 1:1,000 Tween 20) on an ethanol-washed 22 × 40 mm coverslip. A volume of 5 to 10 μL of dissected gonads was then pipetted onto a new ethanol-washed 22 × 40 mm coverslip, and 50 μL of spreading solution (for 1 coverslip, 50 μL: 32 μL of Fixative (4% w/v Paraformaldehyde and 3.2% w/v Sucrose in water), 16 μL of Lipsol solution (1% v/v/ Lipsol in water), and 2 μL of Sarcosyl solution (1% w/v of Sarcosyl in water)) was added, and gonads were immediately distributed over the coverslip using a pipette tip. Coverslips were left to dry at room temperature followed by incubation at 37°C for 1 hour. Coverslips were either processed for staining and expansion or stored at −80°C. For each component analyzed, centrioles stem from a single experiment with approximately 1,000 animals and therefore several hundred thousands nuclei, so that each centriole imaged very likely stems from a different animal.

### Immunofluorescence

Dried coverslips were incubated for 20 minutes in 100% methanol at −20°C. After washing 3 times for 5 minutes in PBS-T (1× PBS, 1:1,000 Tween 20), coverslips were blocked for 20 minutes in 3% w/v BSA in PBS-T at room temperature. Primary antibody incubation was done overnight at room temperature in a moist chamber at 4°C with primary antibodies diluted in 3% w/v BSA in PBS-T supplemented with 0.05% w/v NaN3. Thereafter, coverslips were washed 3 times 5 minutes in PBST prior to incubation with secondary antibodies for 2 hours at room temperature. After three 5-minute washes in PBS-T, the coverslips were mounted on a slide using approximately 20 μL Vectashield (Vector) and sealed with nail polish.

### Ultrastructure expansion microscopy

Dried coverslips were incubated for 20 minutes in 100% methanol at −20°C and washed 3 times in PBS-T for 5 minutes, followed by 2 washes in PBS for 5 minutes each. Coverslips were incubated in a 5-cm Petri dish overnight at room temperature in Acrylamide/Formaldehyde solution (1% Acrylamide and 1% Formaldehyde in PBS) under mild agitation. Thereafter, coverslips were washed 3 times 5 minutes in PBS. For gelation, coverslips were incubated in 50 μl monomer solution (19% (wt/wt) Sodium Acrylate, 10% (wt/wt) Acrylamide, 0.05% (wt/wt) BIS in PBS) supplemented with 0.5% Tetramethylethylenediamine (TEMED), and 0.5% Amonium Persulfate (APS) on a piece of Parafilm for 1 hour at 37°C in a moist chamber in the dark. All subsequent steps were carried out under mild agitation at room temperature unless otherwise stated. Gels were incubated for 15 minutes in denaturation buffer (200 mM SDS, 200 mM NaCl and 50 mM Tris in distilled water, pH = 9) in 5 cm Petri dishes followed by incubation for 1 hour on a 95°C hot plate in fresh denaturation buffer. Gels were transferred to 15 cm Petri dishes washed with distilled water 5 times for 20 minutes, followed by incubation in distilled water overnight at 4°C. The expansion factor was estimated by measuring the gel size with a ruler.

### Immunofluorescence of expanded gels

After expansion, gels were cut in pieces fitting into a 5 cm Petri dish. Prior to staining, gels were blocked for 1 h in blocking buffer (10mM HEPES (pH = 7.4), 3% BSA, 0.1% Tween 20, sodium azide (0.05%)), followed by incubation overnight with primary antibodies diluted in blocking buffer. Gels were washed 3 times in blocking buffer for 10 minutes each, before incubation with secondary antibodies diluted in blocking buffer (supplemented with 0.7 ug/L Hoechst) at 37°C in the dark for 3 hours. Gels were washed 3 times in blocking buffer for 10 minutes before transfer into a 10-cm Petri dish for re-expansion by washing 6 times 20 minutes in distilled water. For imaging, gels were cut and mounted on a 60 × 24 mm coverslip coated with poly-D-lysine (Sigma, #P1024) diluted in water (2 mg/ml) and supported on both longitudinal sides with capillaries attached with superglue. To prevent drying, the edges of the gel were covered with VaLaP (1:1:1 mixture of petroleum:jelly:lanolin:paraffin wax) and the gel was covered with Halocarbon oil 700 for imaging.

### Antibodies used in this study

Primary antibodies raised in rabbit: SAS-6 (1:1,000; [43]), SAS-4 (1:800; [19]), SAS-5 (1:50; [58]), α-tubulin EP1332Y (1:500, Abcam, ab52866), GFP (1:250, a gift from Viesturs Simanis), SPD-5 (1:250; [24]) TBG-1 (1:500; [28]), tagRFP (1:500, Evrogen, AB232), phospho-histone H3 (ser10) (1:300, Merck, 06–570), and mCherry (1:500, Thermo Fisher, PA5-34974).

Primary antibodies raised in mouse: GFP (1:100, Merck, MAB 3580) and FLAG (1:500, Thermo Fisher, MA1-91878).

Primary antibodies raised in rat: tyrosine α-tubulin (EMD Millipore, MAB1864).

Secondary antibodies (all used at 1:1,000): donkey anti-rabbit conjugated to Alexa Fluor 594 (Abcam, ab150072), donkey anti-mouse conjugated to Alexa Fluor 594 (Abcam, ab150112072), goat anti-rat conjugated to Alexa Fluor 594 (Thermo Fisher, A11007), goat anti-rabbit conjugated to Alexa Fluor 488 (Thermo Fisher, A11034), goat anti-mouse conjugated to Alexa Fluor 488 (Thermo Fisher, A11001), donkey anti-rat conjugated to Alexa Fluor 488 (Invitrogen, A21208), and goat anti-rabbit Alexa Fluor 647 (Thermo Fisher, A10523).

### Imaging

2D-STED images were acquired on a Leica TCS SP8 STED 3X microscope with a 100 × 1.4 NA oil-immersion objective, using 488 nm and 589 nm excitation, and 592 nm and 775 nm pulsed lasers for depletion. One pixel Gaussian blur was applied to all images for analysis and display. For display, brightness and contrast were adjusted in the individual channels.

3D-SIM images were acquired on an inverted Nikon Eclipse Ti instrument, with motorized stage and HXP illumination using an APO TIRF 100 × 1.49 NA oil-immersion objective. Image reconstruction was performed with the NIS Elements software and SUM-intensity projected for analysis and display.

Wide-field imaging was performed with a 100×/1.4 Plan-Apochromat objective on a Zeiss Axioplan 2 equipped with a motorized Z-drive (Z steps were 250 nm to 500 nm) and a CoolSnap ES2 camera.

### Determination of effective resolution

The resolution of STED images was determined with 589 nm excitation and depletion with the 775 nm pulsed depletion laser in 10 raw images of α-tubulin using the ImageJ plugin “ImageDecorrelationAnalysis” [65], which resulted in a resolution estimate of 73.4 (± 7.96) nm. This resolution was divided by the average expansion factor of 5.2, determined by the perimeter of α-tubulin signals in the U-Ex-STED images divided by the perimeter of microtubules in EM images. SDs of all 3 measurements (estimation of resolution in the 10 images, measurements for perimeters of α-tubulin, and measurements of perimeters of microtubules) were summed up as a percentage of each individual measurement.

### CLEM analysis

Gonads of genotype *sas-7(or1940[gfp*::*sas-7])III; glo-1(zu931)X; itIs37[pie-1p*::*mCherry*::*H2B*, unc-119(+)] or *ltSi202[pVV103/ pOD1021; Pspd-2*::*GFP*::*SPD-5 RNAiresistant;cb-unc-119(+)]II; sas-7(is1[tagRFP*::*sas-7+loxP])III; glo-1(zu931)X* were dissected in sperm buffer (50 mM Hepes (pH 7.0), 50 mM NACL, 25 mM KCL, 5 mM CaCl2, 1 mM MgSO4, 50 mM Glucose, 1 mg/ml BSA) and transferred on poly-lysine-coated MatTek glass bottom dishes. 3D imaging of gonads was performed using a Nikon Ti2-E epifluorescent microscope equipped with an Andor Zyla-4.2P-CL10 camera before and after an approximately 2-hour and 30-minute fixation at room temperature in 2.2% glutaraldehyde, 0.9% Paraformaldehyde in Cacodylate buffer 0.05M (pH 7.4), 0.09 M sucrose, and 0.9 mM MgCl2. Briefly, specimens were postfixed in 1% osmium tetroxide, 0.8% potassium ferrocyanide in cacodylate buffer (0.1 M, pH 7.2), treated with 0.2% Tannic Acid in 0.05 M cocadylate buffer (pH 7.0), stained with 1% uranyl acetate in Sodium Acetate (pH 5.2), dehydrated in an alcohol series, and embedded in Hard EPON. At 23,000× magnification, 50-nm sections were imaged using a TecnaiSpirit (FEI Company) operated at 80 kV and equipped with an Eagle CCD camera (FEI Company). Using relative positioning of centrioles and nuclei in fluorescence images facilitated the search of centrioles and restricted the number of sections to be imaged. Gaussian blur filtering 1.5 was applied on displayed EM images.

Ultrastructural compartments of the centriole were measured manually using Fiji [66]. Each data point is the average of 4 measurements extracted from lines drawn along the height of the feature. In some cases, ultrastructural compartments could not be measured because they were not visualized accurately, or the view of the centriole was too tilted. Graphs were generated using PlotsofData [67] and SuperPlotsofdata [68]. Procentriole positioning relative to the centriole in S3 Fig was qualitatively assessed on side views, excluding views that were too tilted.

### Electron tomography

Tilt-series from cryo-fixed sections were acquired on a Tecnai F20 operated at 200 kV (Thermo Fischer Scientific using Thermo Scientific Tomography software in continuous tilt scheme from −60° to +60° in 2° steps at −2.5 μm defocus. Data were recorded with a Falcon III DD camera (Thermo Fisher Scientific) in linear mode at 2,000× magnification, corresponding to a pixel size of 3.49. Particles were picked from individual tilt images and 2D Class averages were calculated using Relion [69], Xmipp [70], and Eman2 within the Scipion3 [71] framework. Tilt series alignment and tomogram reconstruction were conducted using EMAN 2.9 [72]. Tomogram subvolumes for the detection of ribosome-free area were extracted using Imod 4.9 [73] and maximum intensity project in Fiji [66].

### Worm strains used in this study

- N2 (Bristol)
- TMD101: *pcmd-1(t3421); mikSi6[pmai-2*:*GFP*::*C17D12*.*7] II* [21]
- TMD117: *pcmd-1(t3421); mikSi9[pmai-2*:*C17D12*.*7*::*GFP] II* [21]
- DAM276: *ltSi40 [pOD1227; Psas-6*::*sas-6reencoded*::*GFP; cb unc-119(+)] II; sas-6(ok2554) IV* [57]
- GZ1934: *sas-1(is7[3xflag*::*sas-1]) III* (this study)
- GZ1966: *sas-1(is6[sas-1*::*3xflag]) III* (this study)
- OC994: *sas-4(bs195[sas-4*::*gfp] III* (a gift from Kevin O’Connell)
- DAM307: *vieSi16[pAD390; Phyls1*:*mcherry*::*hyls-1; cb unc-119(+)] IV* [60]
- GZ1528: *spd-2(is2[tagRFP*::*spd-2 +loxP]) I; sas-7(or1940(gfp*::*sas-7)) III; glo-1(zu931) X* (this study)
- DAM640: *spd-2(vie4[spd-2*::*gfp +loxP]) I* [61]
- JLF375: *mzt-1(wow51[GFP*:*MZT-1]) I; zif-1(gk117) III* [27]
- GZ1929: *sas-6(is10[3xflag*::*sas-6]) IV* (this study)

## Supporting information

S1 Fig Procentriole composition and maturation. (A) Widefield image of 2 pairs of centriole/procentriole in an S-phase cell located in the mitotic zone of the gonad after U-Ex, stained for SAS-6 and α-tubulin. Note that procentrioles harbor SAS-6 but no α-tubulin at this stage. (B) U-Ex-STED images of centrioles from the mitotic zone (left and middle) and meiotic prophase (right). Images illustrate that the amount of ZYG-1 on the centriole (but not on the procentriole) varies: During meiotic prophase, ZYG-1 levels on the centriole are very low. For quantification, a line was drawn from the center of the centriole to the outside of the procentriole and the intensity profile along this line measured, as represented by the dashed arrows. (Left and middle) In the mitotic zone, whereas 12/25 centrioles exhibited a similar distribution to that observed in the vast majority of meiotic prophase centrioles (see right), 13/25 displayed the ZYG-1 signal more prominently than during meiotic prophase, with 2 clear ZYG-1 peaks, one at the base of the procentriole and one in the middle of the centriolar SAS-6::GFP signal. (Right) In 95% of meiotic prophase centrioles (19/20), a single ZYG-1 signal intensity peak was detected outside of the centriole peak, directly under the procentriole. (C) 3D-SIM sum intensity Z-projected image of a nucleus in the mitotic zone of an expanded gonad. Phosphorylated Histone 3 marks nuclei in mitosis. Insets on the right show that all 4 centrioles contain α-tubulin, unlike in S or G2 phase. SIM, Structured Illumination Microscopy; U-Ex-STED, Ultrastructure Expansion coupled with STimulated Emission Depletion.
(PDF)

S2 Fig Schematic of 9-fold symmetrization process. Images of centrioles seen from the top were centered in a square ROI, then cropped and iteratively rotated by 40° (left). The resulting nine images were arranged in a stack and sum intensity projected (middle). The resulting image represents a 9-fold symmetrized image (right).
(PDF)

S3 Fig Procentriole position along mother centrioles. (A) (Left) Example of an EM side view where the inner tube is visible as 2 parallel noncontinuous lines. (Middle) Overlay of schematics with the EM image. Note that the procentriole is not present in this particular section. (B, C) (Left) EM side views of early meiotic prophase centrioles with centered procentriole (B) and off-centered procentriole (C). (Middle) Overlay of schematics with EM images. (Right) Corresponding schematic representations. Paddlewheels are highlighted in blue, microtubules in red, and procentrioles in yellow. Dashed lines indicate the middle of the height of centrioles (blue) and procentrioles (yellow). (D) U-Ex STED images illustrating that the procentriole can grow from different positions along the side of the centriole. Pink dashed lines indicate bottom and top of the centriole, white dashed lines the edges of the procentriole. Note that the procentrioles can emanate from the center of a short centriole (left), from the center of a higher centriole (middle) or from closer to one end of the centriole (right). Data underlying the graphs shown in the figure can be found in S1 Data. EM, electron microscopy; U-Ex-STED, Ultrastructure Expansion coupled with STimulated Emission Depletion.
(PDF)

S4 Fig Schematic of U-Ex and EM images image overlay. In EM and U-Ex-STED top views, centrioles with slightly tilted orientations were circularized with the Fiji plugin “Transform-Interactive Affine”. The grey levels of the EM image were inverted, and the circularized EM and U-Ex-STED images then 9-fold symmetrized as illustrated in S2 Fig. The perimeters of the microtubule wall in symmetrized EM images and of the α-tubulin signal in symmetrized U-Ex-STED images were measured, and the symmetrized U-Ex-STED image adjusted in dimensions so that the α-tubulin signal had the same perimeter as the microtubule wall in symmetrized EM. To overlay symmetrized images, U-Ex-STED images were rotated so that individual α-tubulin signals perfectly overlapped with individual microtubule signals in symmetrized EM images. Thereafter, images were overlayed in individual color channels. The rotational angles and size adjustments applied for symmetrized images were then applied also to the raw nonsymmetrized images (indicated by the dashed arrows), which were then treated likewise. EM, electron microscopy; U-Ex-STED, Ultrastructure Expansion coupled with STimulated Emission Depletion.
(PDF)

S5 Fig ET class averaging of individual microtubules reveals novel densities. (A) 628 particles containing microtubules and paddlewheels were picked from individual ET tilt series of 4 centrioles. Class-averaging resulted in 12 classes, 3 of which were well defined and together contained 92% of input particles (classes 1–3, colorized with the LUT “Fire”, low intensities in blue, high intensities in magenta and red). (B) Line intensity profile of classes 1–3 along the lines indicated in (A) (10 pixels wide). The microtubule displayed consistently more density on the side located under the paddlewheel (“A”) than on the other side (“B”). (C) Overlay of U-Ex-STED images for FLAG::SAS-1, SPD-5 and microtubules, together with EM images (inverted grey levels) of centrioles from early meiotic prophase. Circularized images (left two panels), corresponding 9-fold symmetrized versions (next two panels), and magnification of the insets highlighted by the white box (very right). EM, electron microscopy; ET, electron tomography; U-Ex-STED, Ultrastructure Expansion coupled with STimulated Emission Depletion.
(PDF)

S1 Data Excel file containing datapoints for Figs 2D, 2E, 3C, 4C, 4D–4K, 5B, 5C, and S3. (XLSX)

## Abbreviations

CLEM: Correlative Light Electron Microscopy
EM: electron microscopy
ET: electron tomography
IF: immunofluorescence
IPD: Inter Paddlewheel Density
PCM: PeriCentriolar Material
SCD: SAS-6/4/1 Containing Density
SIM: Structured Illumination Microscopy
U-Ex-STED: Ultrastructure Expansion coupled with STimulated Emission Depletion
