# Supplementary figures and images for: Molecular architecture of the C. elegans centriole
Source: PLoS Biol. 2022 Sep 15;20(9):e3001784. doi: 10.1371/journal.pbio.3001784 (PMC9531800; doi:10.1371/journal.pbio.3001784)

## A Expanded, wide-field

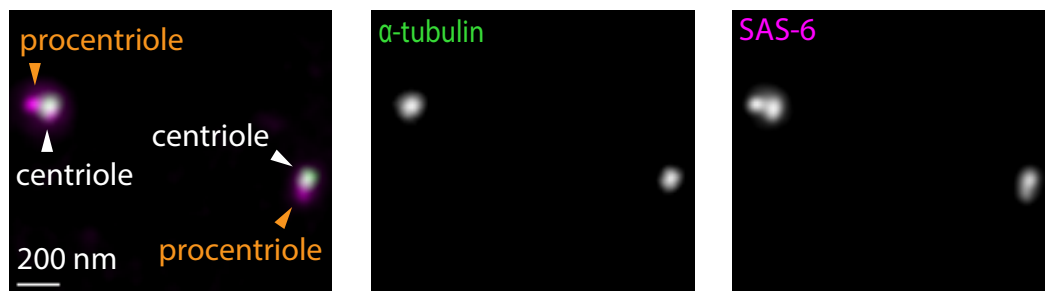

## B U-Ex STED

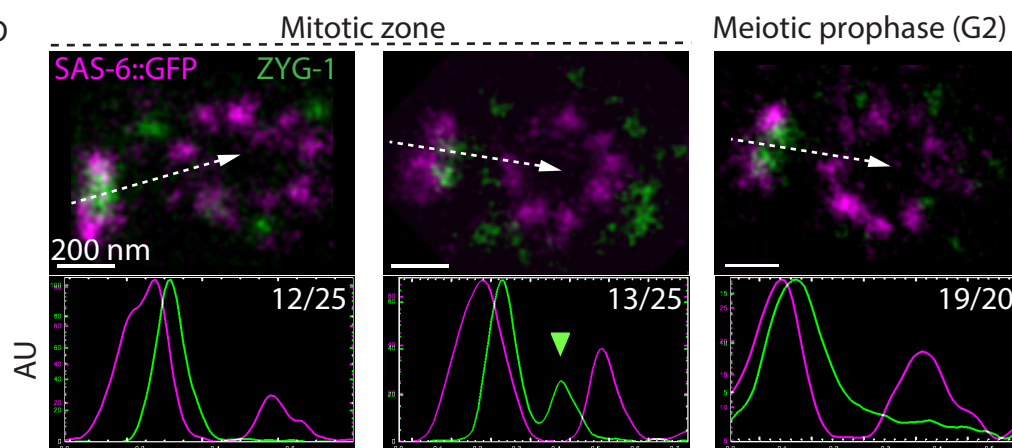

## C Mitotic zone, 3D-SIM

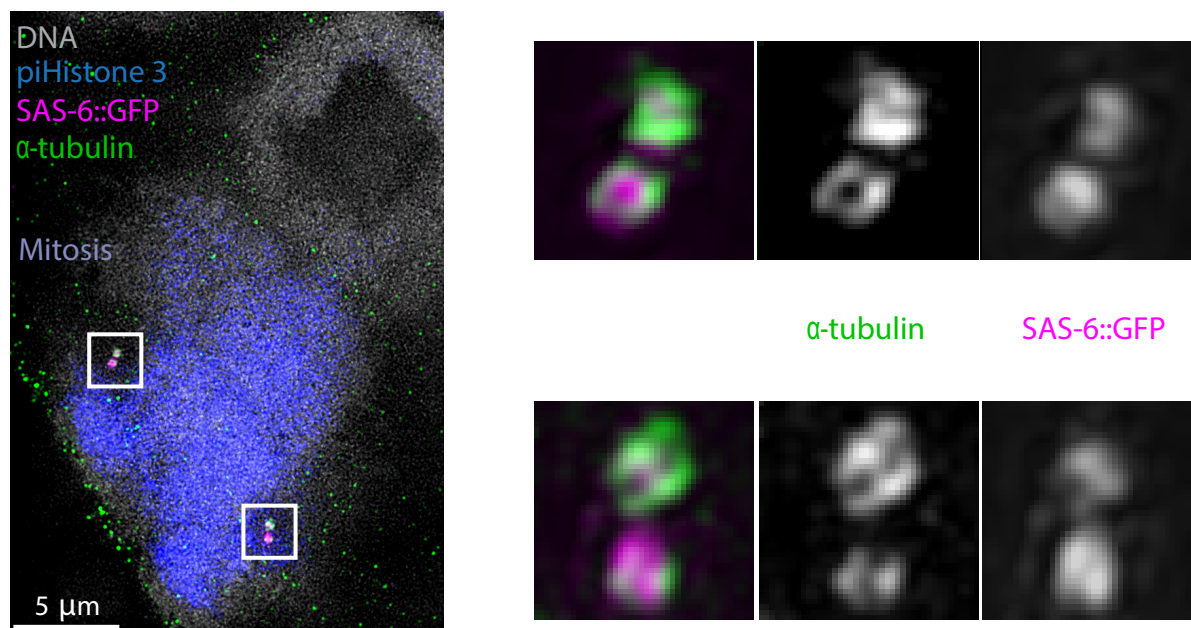

Figure S1

Supplement: S1 Fig — (A) Widefield image of 2 pairs of centriole/procentriole in an S-phase cell located in the mitotic zone of the gonad after U-Ex, stained for SAS-6 and α-tubulin. Note that procentrioles harbor SAS-6 but no α-tubulin at this stage. (B) U-Ex-STED images of centrioles from the mitotic zone (left and middle) and meiotic prophase (right). Images illustrate that the amount of ZYG-1 on the centriole (but not on the procentriole) varies: During meiotic prophase, ZYG-1 levels on the centriole are very low. For quantification, a line was drawn from the center of the centriole to the outside of the procentriole and the intensity profile along this line measured, as represented by the dashed arrows. (Left and middle) In the mitotic zone, whereas 12/25 centrioles exhibited a similar distribution to that observed in the vast majority of meiotic prophase centrioles (see right), 13/25 displayed the ZYG-1 signal more prominently than during meiotic prophase, with 2 clear ZYG-1 peaks, one at the base of the procentriole and one in the middle of the centriolar SAS-6::GFP signal. (Right) In 95% of meiotic prophase centrioles (19/20), a single ZYG-1 signal intensity peak was detected outside of the centriole peak, directly under the procentriole. (C) 3D-SIM sum intensity Z-projected image of a nucleus in the mitotic zone of an expanded gonad. Phosphorylated Histone 3 marks nuclei in mitosis. Insets on the right show that all 4 centrioles contain α-tubulin, unlike in S or G2 phase. SIM, Structured Illumination Microscopy; U-Ex-STED, Ultrastructure Expansion coupled with STimulated Emission Depletion. (PDF) [file pbio.3001784.s001.pdf]

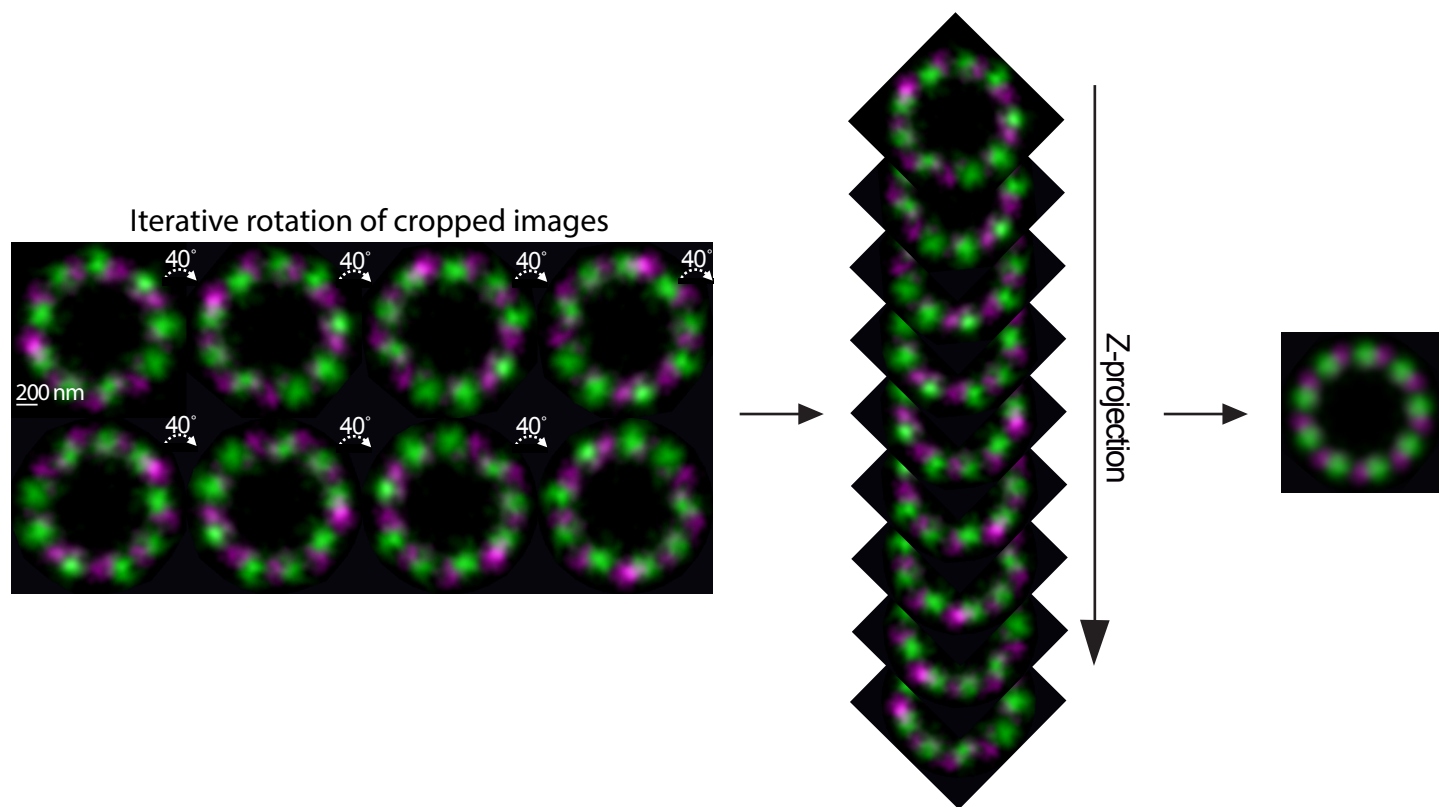

Figure S2

Supplement: S2 Fig — Images of centrioles seen from the top were centered in a square ROI, then cropped and iteratively rotated by 40° (left). The resulting nine images were arranged in a stack and sum intensity projected (middle). The resulting image represents a 9-fold symmetrized image (right). (PDF) [file pbio.3001784.s002.pdf]

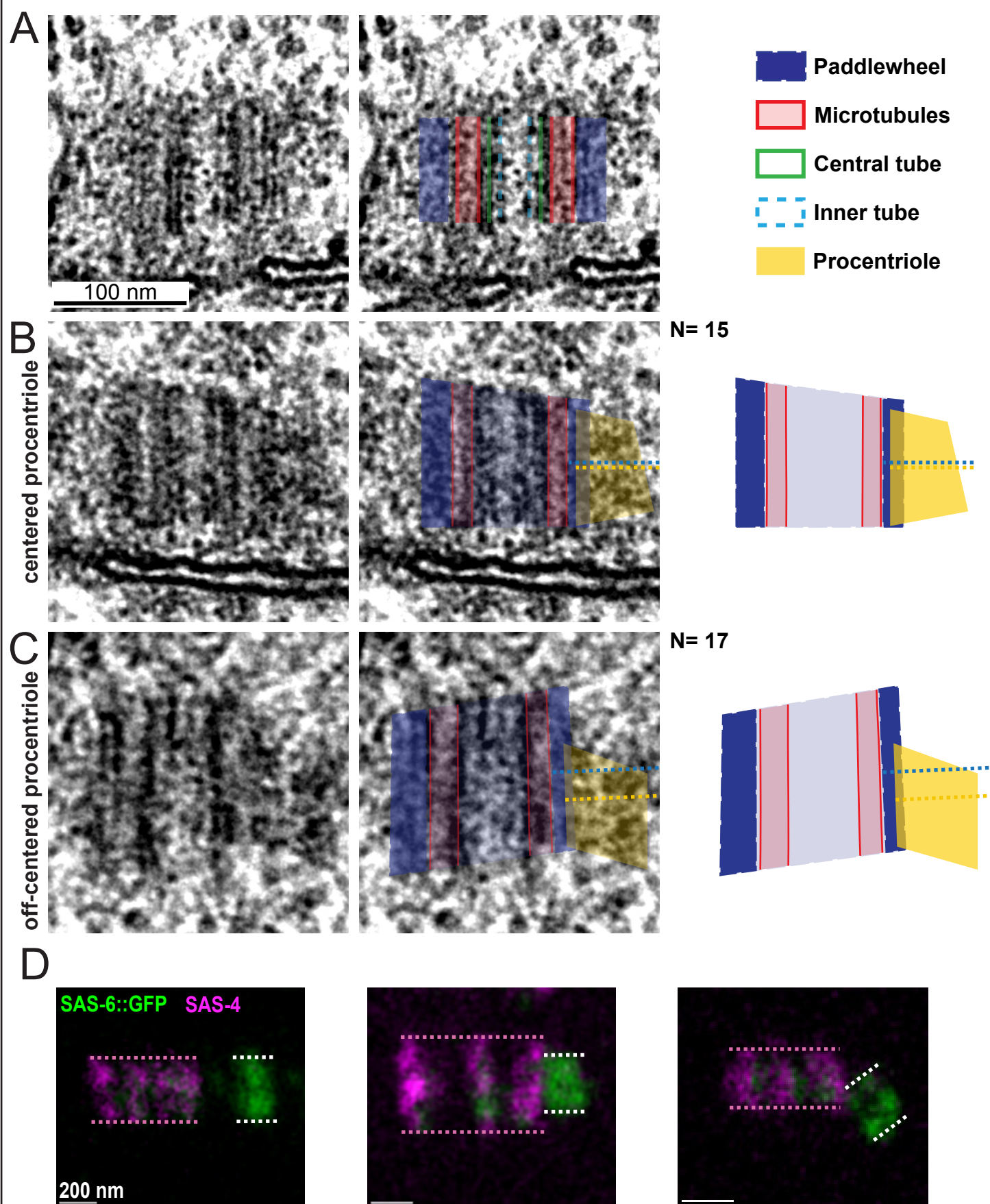

Figure S3

Supplement: S3 Fig — (A) (Left) Example of an EM side view where the inner tube is visible as 2 parallel noncontinuous lines. (Middle) Overlay of schematics with the EM image. Note that the procentriole is not present in this particular section. (B, C) (Left) EM side views of early meiotic prophase centrioles with centered procentriole (B) and off-centered procentriole (C). (Middle) Overlay of schematics with EM images. (Right) Corresponding schematic representations. Paddlewheels are highlighted in blue, microtubules in red, and procentrioles in yellow. Dashed lines indicate the middle of the height of centrioles (blue) and procentrioles (yellow). (D) U-Ex STED images illustrating that the procentriole can grow from different positions along the side of the centriole. Pink dashed lines indicate bottom and top of the centriole, white dashed lines the edges of the procentriole. Note that the procentrioles can emanate from the center of a short centriole (left), from the center of a higher centriole (middle) or from closer to one end of the centriole (right). Data underlying the graphs shown in the figure can be found in S1 Data. EM, electron microscopy; U-Ex-STED, Ultrastructure Expansion coupled with STimulated Emission Depletion. (PDF) [file pbio.3001784.s003.pdf]

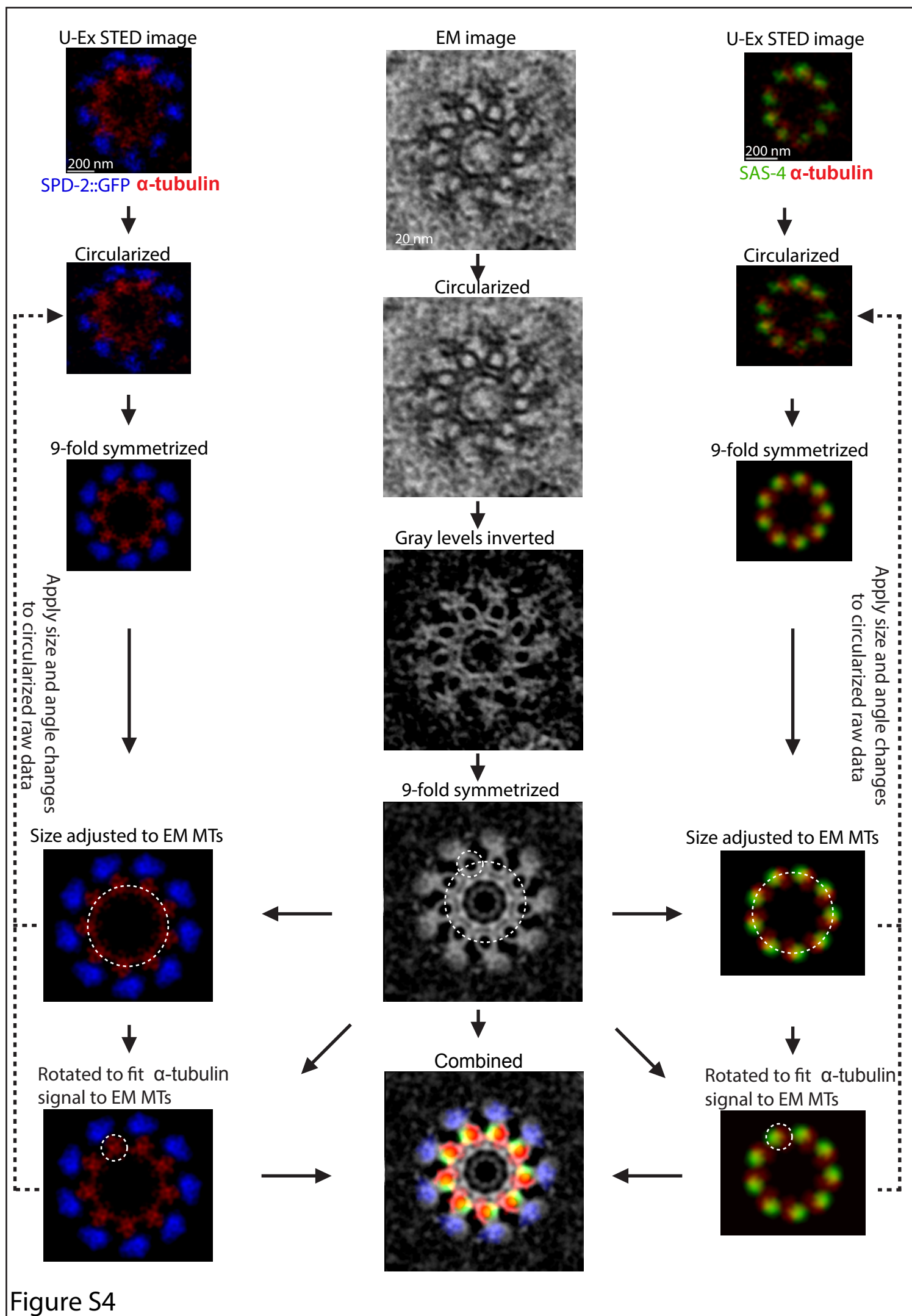

Figure S4

Supplement: S4 Fig — In EM and U-Ex-STED top views, centrioles with slightly tilted orientations were circularized with the Fiji plugin “Transform-Interactive Affine”. The grey levels of the EM image were inverted, and the circularized EM and U-Ex-STED images then 9-fold symmetrized as illustrated in S2 Fig. The perimeters of the microtubule wall in symmetrized EM images and of the α-tubulin signal in symmetrized U-Ex-STED images were measured, and the symmetrized U-Ex-STED image adjusted in dimensions so that the α-tubulin signal had the same perimeter as the microtubule wall in symmetrized EM. To overlay symmetrized images, U-Ex-STED images were rotated so that individual α-tubulin signals perfectly overlapped with individual microtubule signals in symmetrized EM images. Thereafter, images were overlayed in individual color channels. The rotational angles and size adjustments applied for symmetrized images were then applied also to the raw nonsymmetrized images (indicated by the dashed arrows), which were then treated likewise. EM, electron microscopy; U-Ex-STED, Ultrastructure Expansion coupled with STimulated Emission Depletion. (PDF) [file pbio.3001784.s004.pdf]

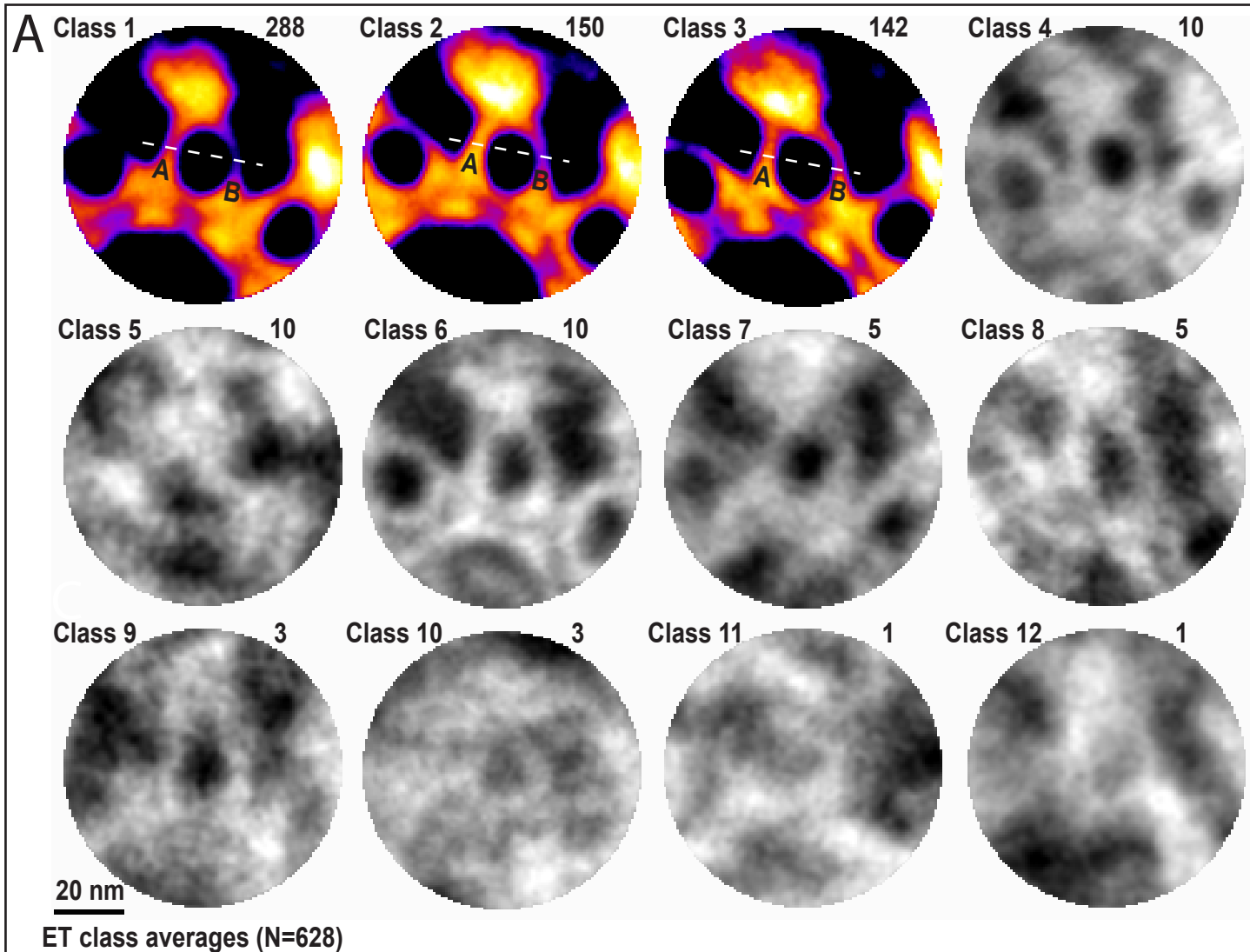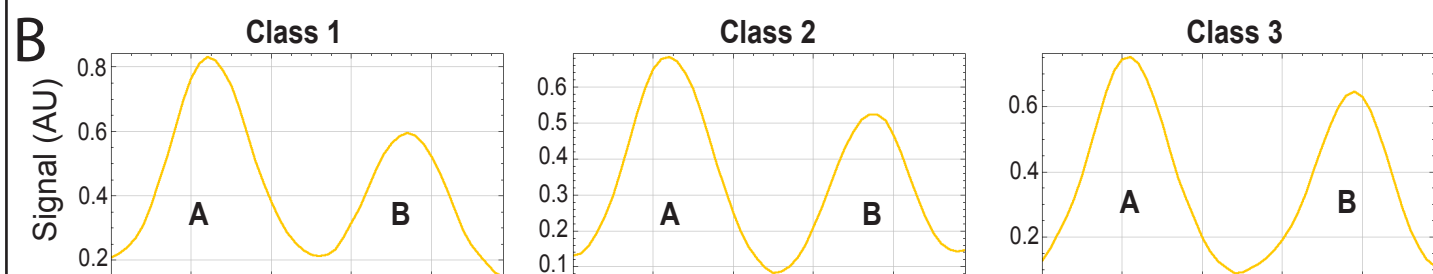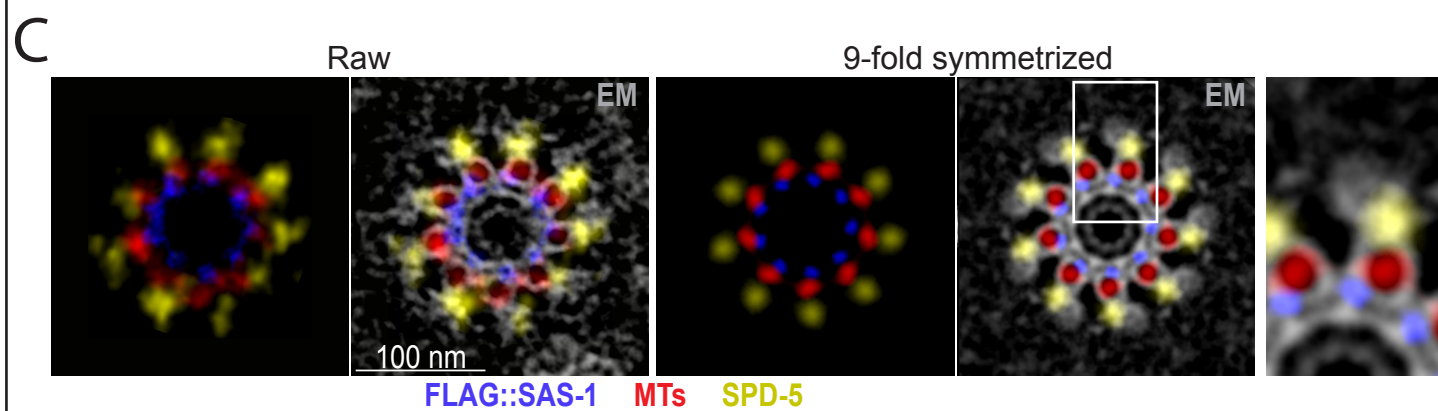

Figure S5

Supplement: S5 Fig — (A) 628 particles containing microtubules and paddlewheels were picked from individual ET tilt series of 4 centrioles. Class-averaging resulted in 12 classes, 3 of which were well defined and together contained 92% of input particles (classes 1–3, colorized with the LUT “Fire”, low intensities in blue, high intensities in magenta and red). (B) Line intensity profile of classes 1–3 along the lines indicated in (A) (10 pixels wide). The microtubule displayed consistently more density on the side located under the paddlewheel (“A”) than on the other side (“B”). (C) Overlay of U-Ex-STED images for FLAG::SAS-1, SPD-5 and microtubules, together with EM images (inverted grey levels) of centrioles from early meiotic prophase. Circularized images (left two panels), corresponding 9-fold symmetrized versions (next two panels), and magnification of the insets highlighted by the white box (very right). EM, electron microscopy; ET, electron tomography; U-Ex-STED, Ultrastructure Expansion coupled with STimulated Emission Depletion. (PDF) [file pbio.3001784.s005.pdf]
