# Supplementary material for: Molecular architecture of the C. elegans centriole
Source: PLoS Biol. 2022 Sep 15;20(9):e3001784. doi: 10.1371/journal.pbio.3001784 (PMC9531800; doi:10.1371/journal.pbio.3001784)
Supplement: Attachment — Submitted filename: 126915_1_rebuttal_2397858_r777x3.pdf [file pbio.3001784.s007.pdf]

Revision Plan

Review COMMONS

Manuscript number: RC-2022-01466

Corresponding author(s): Pierre, Gönczy

[The "revision plan" should delineate the revisions that authors intend to carry out in response to the points raised by the referees. It also provides the authors with the opportunity to explain their view of the paper and of the referee reports.

The document is important for the editors of affiliate journals when they make a first decision on the transferred manuscript. It will also be useful to readers of the reprint and help them to obtain a balanced view of the paper.

If you wish to submit a full revision, please use our "Full Revision" template. ***It is important to use the appropriate template to clearly inform the editors of your intentions.****]*

1. General Statements [optional]

This section is optional. Insert here any general statements you wish to make about the goal of the study or about the reviews.

2. Description of the planned revisions

We delineate hereafter the steps that we will take to address in full the minor comments made by the three reviewers.

Reviewer #1 (Evidence, reproducibility and clarity (Required)):

The authors analyze here the organization of centrioles in C. elegans, by combining the physical expansion of the specimens (by about 5-fold) with stimulated emission depletion (STED) microscopy. They analyze a large number of centriole components in different experiments, and they combine the data into a convincing model of the centriole, which is presented in conjunction with electron microscopy images of this structure.
The work is solid, well-performed and technically sound. While this reviewer is not a centriole expert, the work also appears to be sufficiently novel, simply due to its precision, to warrant publication.

Reviewer #1 (Significance (Required)):

I only have one suggestion, which the authors may consider. Most of their work involves analyzing the symmetry of the structures, as presented, for example, in Fig. 4. However, symmetry problems, observable in individual structures, may also be informative. Are specific

proteins more prone to variable localization, as, for example, SPD-2-C or SPD-5, while others are more stereotypically organized? Could an analysis of the variability of the stainings provide information on flexibility in the centriole organization?
> One cannot compare in a quantitative manner the distribution variability between components, unfortunately, since each component is analyzed with distinct reagents (i.e. antibodies and fluorescent fusion proteins). As a result, the variability due to experimental noise cannot be distinguished from that stemming from potential biological differences. Note, however, that the variability of ring diameters of each component is reported in Figure 3C.

Reviewer #2 (Evidence, reproducibility and clarity (Required)):

In this manuscript, Woglar et al describe molecular features of the C. elegans centriole with unprecedented detail. By adapting U-ExM to extracted gonads and combining it with EM and TEM data, the authors precisely mapped the location of 12 components. They uncovered that these centrioles are shorter than in the embryo, have the same structural elements, and show an offset of centriolar proteins distribution relative to microtubules which results in chirality. Their detailed analysis also identified two novel electron-dense regions: the Inter Paddlewheel Density (IPD); and the SAS-6/4/1 Containing Density (SCD). This manuscript is a very nice description of C. elegans centrioles and we have mostly minor comments to improve it.

1. Regarding the duplication and maturation section, the authors state in the abstract: "We uncovered that the procentriole assembles from a location on the centriole margin characterized by SPD-2 and ZYG-1 accumulation.". The data collected by the authors do not provide evidence of enrichment of ZYG-1 and SDP-2 prior to procentriole assembly (in the main text the authors clearly say they are speculating). This statement in the abstract should be corrected to more accurately match what is described in the main text and supported by the results.
> We did not mean to imply that the enrichment of ZYG-1 and SPD-2 occurs prior to procentriole assembly, and will rectify the wording in the abstract to clarify this point.

2. It is stated in the main text that the procentrioles can emanate from the middle of the centriole but no representative image is shown (only shown for off-centered procentrioles or very short templates). It is also referred that this may have implications on chirality- it would be important to explain better those implications, as well as offer an example of this configuration.
> To clarify the point regarding the procentriole emanating from the middle of the centriole, we will go through the EM data set anew and adapt text plus figure as appropriate. In addition, we intend to complement the EM data with new STED images to strengthen this point. We will also work on the writing to better explain the implications of our findings for organelle chirality.

3. The authors mention "core PCM" throughout the manuscript without explaining or referencing its definition. Would be useful to the reader if more information is provided.
> We will rectify the text to better explain what is meant by this term, i.e. the interphase PCM, prior to PCM maturation. In fact, the term “PCM core” was coined by Erpf and co-workers in

2019 (https://doi.org/10.1016/j.cub.2019.03.029), and we will reference their work, using this exact wording in the revised manuscript for clarity.

4. FigS1.A looks strange because procentrioles seem much longer than centrioles and their relative orientation does not seem to be orthogonal. If this image is representative, it would be helpful to have a diagram explaining the image.
> We realize that this particular image may have led to confusion, as SAS-6 marks not only the two procentrioles (in side views) but also the two centrioles (in top views); this may have led to the impression that the procentrioles are longer. Therefore, we will replace this image by another representative picture where the procentrioles are slightly further apart from one another. See also point 3 of reviewer #3.

5. In the main text it is said: "Four components were found to localize to the paddlewheel: HYLS-1[N], SPD-2, SPD-5 and PCMD-1." and SDP-5 is represented in the final scheme (Fig. 7). However, an overlay of SPD5 and EM data is never shown. The authors may extrapolate that SPD-5 localizes there because it is interior to SPD-2 with no offset compared to α-tubulin, but if this is the case it should be clearer in the text.
> In response to this request, we will provide an additional Supplementary figure panel with the overlay between the EM data and SPD-5, as well as with SAS-1, which was likewise not reported in the initial submission merely because of space considerations.

6. A statistics section is missing in which the program used is detailed and whether the {plus minus} values in the figures depict SD or SEM. The number of independent experiments should also be mentioned.
> A two-tailed Student's t-test was utilized for Figure 2C, as spelled out in the figure legend. Likewise, the legend of Figures 3C and 5C spell out what the boxplots correspond to, whereas we forgot to mention in the legend of Figures 2A that the +/- values correspond to SD; evidently, this will be rectified in the revised manuscript. We will also spell out explicitly in the Materials and Methods section that the centrioles analyzed for each component stem from an experiment with hundreds of animals.

7. Although symmetrization has been increasingly adopted by the field, it would still be useful to reference previous examples of its application in centriole structure analysis.
> We will quote the original 1970 work of Friedman (doi: 10.1016/s0022-5320(70)80003-x) regarding symmetrization of EM images, as well as a recent application of this method for ultrastructural analysis of centrioles (Bezler et al., 2022; doi: 10.1091/mbc.E22-04-0123).

8. S1B and S1C figure labels are swapped.
>Apologies about this mistake, which will be fixed.

9. The authors claim that "the procentriole likewise harbors little SAS-4 initially and that more protein is recruited at prometaphase, resulting in similar levels of SAS-4 in the centriole and the procentriole by then (Fig. 2D)". Can the authors provide some sort of semi-quantitative readout?

> The quantification of the initial levels of SAS-4 at the procentriole is reported in Figure 2A, and we will provide an assessment of prometaphase levels by quantifying the images in Figure 2D.

10. In Figure 5A side view, the presence of an inner tube is not very clear. Given that diameter quantifications were done using the mostly side views, it would be beneficial if the authors could provide a clearer image.
> The side view in Figure 5A was chosen because it displays most of the ultrastructural features in a favorable manner, but we realize that this is not the most telling image for the inner tube. To address this issue, we will go through the EM data set anew and assess whether a suitable example can be shown to complement the current image. Moreover, we will curate the EM data anew to remove from the data set those side views in which inner tube diameter appears uncertain.

Reviewer #2 (Significance (Required)):

Overall, these observations contribute toward a better understanding of centriole structure, molecular composition and diversity, with a particular focus on C. elegans. The precision of the approach developed by the authors (U-ExM and EM overlay) is a valuable tool and will be of interest to the centriole biology field and to cell biologists in general.
Reviewer expertise: Cellular and molecular biologists working in the field of centrioles.

Reviewer #3 (Evidence, reproducibility and clarity (Required)):

In this manuscript Woglar et al. use several light and electron microscopy techniques combined with averaging/registration methodologies to produce a comprehensive molecular map of the centriole in the C. elegans gonad. The images produced are very impressive and potentially very informative, allowing the authors to draw several important conclusions (e.g. about the chirality of the structure, and the potential organisation of Sas-6 in the cartwheel, the latter of which has been controversial in this species). Thus, although the manuscript is largely descriptive, there is a lot here that will be of great interest to the centriole field. The manuscript is generally well written and well presented, and, although I am not a great expert in all of these techniques, the data seems to solidly support the main conclusions. I therefore have only a small number of relatively minor suggestions for improvements.

Minor Comments:

1. It should be clarified whether the centrioles being examined here are organising genuine PCM and MTs. I know that in the embryo SPD-2 and SPD-5 are considered the main organisers of the mitotic PCM, and these centrioles are in S-phase or G2 (so I'm not sure if they are organising any PCM). SPD-5 is located internally to SPD-2, perhaps suggesting that these centrioles are

not organising a bona fide PCM? On the other hand, TBG-1 and MZT-1 are located at the periphery, so I assume these centrioles are organising MTs?
> In the revised manuscript, we will refer to previous work establishing that centrioles during meiotic prophase do not organize PCM acting as microtubule organizing centers (MTOCs) (Zhou et al., 2009; DOI: 10.1083/jcb.200902101; Mikeladze-Dvali et al., 2012; doi: 10.1242/dev.075440.).

2. I think the labels (A, B, C) in Figure S1 are probably in the wrong order and are not referred to correctly in the main text.
>Apologies about this mistake, which will be fixed.

3. In Figure S1A two centrioles are shown that seem to be touching at their proximal ends, which I initially interpreted as meaning the centrioles were engaged. If so, there seems to be a long tail of Sas-6 connecting the two centrioles that extends well below the centriole MTs. However, reading the legend, I think this interpretation is incorrect, and the images are showing two separate centrioles that just happen to be touching? Perhaps swap in another image that won't lead to this potential confusion?
> We realize that this particular image may have led to confusion, as SAS-6 labels not only the two procentrioles (side views) but also the two centrioles (top views), which may have led to the impression that the procentrioles are longer. Therefore, we will replace this image by another representative picture where the procentrioles are slightly further apart from one another. See also point 4 of reviewer #2.

Reviewer #3 (Significance (Required)):

Although several papers have reported high resolution molecular mapping of centrioles, this one is perhaps the most detailed and does a nice job of superimposing the molecular structures on high quality EM images. Not all of these C. elegans proteins are obviously conserved, but C. elegans is a 'poster-child' model organism for centriole research, and this broad architecture will be of great interest to the entire centriole/centrosome (and also cilia) fields. In addition, the observation of chirality that is intrinsic to the inner centriole structure, and that Sas-6 is likely organised into rings rather than a steep helix, are important conclusions.

I am an expert in centrioles and high resolution imaging, but not EM.

Revision Plan

3. Description of the revisions that have already been incorporated in the transferred manuscript

Please insert a point-by-point reply describing the revisions that were already carried out and included in the transferred manuscript. If no revisions have been carried out yet, please leave this section empty.

4. Description of analyses that authors prefer not to carry out

Please include a point-by-point response explaining why some of the requested data or additional analyses might not be necessary or cannot be provided within the scope of a revision. This can be due to time or resource limitations or in case of disagreement about the necessity of such additional data given the scope of the study. Please leave empty if not applicable.
