# Supplementary material for: Molecular architecture of the C. elegans centriole
Source: PLoS Biol. 2022 Sep 15;20(9):e3001784. doi: 10.1371/journal.pbio.3001784 (PMC9531800; doi:10.1371/journal.pbio.3001784)
Supplement: Attachment — Submitted filename: Woglar_2022_response_to _reviewers.pdf [file pbio.3001784.s008.pdf]

We thank the reviewers for their careful reading and suggestions, which helped us improve the manuscript. We have addressed all the points that have been raised, as detailed below point-by-point. In addition, we have made some minor wording changes to streamline and further clarify the writing.

Reviewer #1 (Evidence, reproducibility and clarity (Required)):

The authors analyze here the organization of centrioles in C. elegans, by combining the physical expansion of the specimens (by about 5-fold) with stimulated emission depletion (STED) microscopy. They analyze a large number of centriole components in different experiments, and they combine the data into a convincing model of the centriole, which is presented in conjunction with electron microscopy images of this structure.
The work is solid, well-performed and technically sound. While this reviewer is not a centriole expert, the work also appears to be sufficiently novel, simply due to its precision, to warrant publication.

Reviewer #1 (Significance (Required)):

I only have one suggestion, which the authors may consider. Most of their work involves analyzing the symmetry of the structures, as presented, for example, in Fig. 4. However, symmetry problems, observable in individual structures, may also be informative. Are specific proteins more prone to variable localization, as, for example, SPD-2-C or SPD-5, while others are more stereotypically organized? Could an analysis of the variability of the stainings provide information on flexibility in the centriole organization?

> One can unfortunately not compare in a quantitative manner the variability in distribution between components, since each of them is analyzed with distinct reagents (i.e. antibodies or fluorescent fusion proteins). As a result, the variability due to experimental noise cannot be distinguished from that stemming from potential biological differences. Note, however, that the variability of ring diameters of each component is reported in Figure 3C.

Reviewer #2 (Evidence, reproducibility and clarity (Required)):

In this manuscript, Woglar et al describe molecular features of the C. elegans centriole with unprecedented detail. By adapting U-ExM to extracted gonads and combining it with EM and TEM data, the authors precisely mapped the location of 12 components. They uncovered that these centrioles are shorter than in the embryo, have the same structural elements, and show an offset of centriolar proteins distribution relative to microtubules which results in chirality. Their detailed analysis also identified two novel electron-dense regions: the Inter Paddlewheel Density (IPD); and the SAS-6/4/1 Containing Density (SCD). This manuscript is a very nice description of C. elegans centrioles and we have mostly minor comments to improve it.

1. Regarding the duplication and maturation section, the authors state in the abstract: "We uncovered that the procentriole assembles from a location on the centriole margin characterized

by SPD-2 and ZYG-1 accumulation.". The data collected by the authors do not provide evidence of enrichment of ZYG-1 and SDP-2 prior to procentriole assembly (in the main text the authors clearly say they are speculating). This statement in the abstract should be corrected to more accurately match what is described in the main text and supported by the results.

> We did not mean to imply that the enrichment of ZYG-1 and SPD-2 occurs prior to procentriole assembly and have rectified the wording in the abstract to clarify this point (p. 2 of the revised manuscript).

2. It is stated in the main text that the procentrioles can emanate from the middle of the centriole, but no representative image is shown (only shown for off-centered procentrioles or very short templates). It is also referred that this may have implications on chirality- it would be important to explain better those implications, as well as offer an example of this configuration.

> Prompted by the important comment of the reviewer, we have conducted further analysis with U-Ex-STED to strengthen this point, resulting in a new Figure S3D. Here, we show instances where the procentriole is centered (either covering the entire side of the centriole or only the central part of it, depending on the height of the centriole) or off-centered, supporting and extending the analysis by EM. Moreover, we clarified in the text what these observations could mean for the onset of procentriole formation along the centriole and discuss better the implications this has on assigning organelle chirality (p. 11 of the revised manuscript).

3. The authors mention "core PCM" throughout the manuscript without explaining or referencing its definition. Would be useful to the reader if more information is provided.

> We now explain better what is meant by this term (i.e. the interphase PCM, prior to PCM maturation, see bottom of page 4). Moreover, instead of “PCM core’, we now use throughout the manuscript the term “core PCM”, which has been coined previously to refer to the same entity (Erpf et al., 2019, doi: org/10.1016/j.cub.2019.03.029).

4. FigS1.A looks strange because procentrioles seem much longer than centrioles and their relative orientation does not seem to be orthogonal. If this image is representative, it would be helpful to have a diagram explaining the image.

> We realize that this particular image may have led to some confusion, as SAS-6 marks not only the two procentrioles in side-views but also the two centrioles in top views, which may have led to the impression that the procentrioles are longer than they actually are. Therefore, we have replaced this image with a new one with two pairs of centrioles located in the mitotic zone, where centrioles are frequently further away from each other. See also point 3 of reviewer #3.

5. In the main text it is said: "Four components were found to localize to the paddlewheel: HYLS-1[N], SPD-2, SPD-5 and PCMD-1." and SDP-5 is represented in the final scheme (Fig. 7). However, an overlay of SPD5 and EM data is never shown. The authors may extrapolate that SPD-5 localizes there because it is interior to SPD-2 with no offset compared to α-tubulin, but if this is the case it should be clearer in the text.

> In response to this request, we provide a new supplementary figure panel (Figure S5C) showing the overlay between the EM data and SPD-5 distribution, as well as with that of SAS-1, which was likewise not reported in the initial submission because of space considerations.

6. A statistics section is missing in which the program used is detailed and whether the {plus minus} values in the figures depict SD or SEM. The number of independent experiments should also be mentioned.

> A two-tailed Student's t-test was utilized for Figure 2C, as spelled out in the figure legend. Likewise, the legend of Figures 3C and 5C do spell out what the boxplots correspond to. However, we forgot to mention in the legend of Figures 2A that the +/- values correspond to SD; this is rectified in the revised manuscript (p. 17 of the revised manuscript). We now also spell out explicitly in the Materials and Methods section that centrioles analyzed for each component stem from an experiment with ~ 1000 animals and therefore several hundred thousands nuclei. As a result, each centriole imaged almost certainly stems from a different animal (p. 28 of the revised manuscript).

7. Although symmetrization has been increasingly adopted by the field, it would still be useful to reference previous examples of its application in centriole structure analysis.

> In the revised manuscript, we quote the original 1970 work of Friedman (doi: 10.1016/s0022-5320(70)80003-x) regarding symmetrization of EM images, as well as a recent application of this method for ultrastructural analysis of centrioles (Bezler et al., 2022; doi: 10.1091/mbc.E22-04-0123).

8. S1B and S1C figure labels are swapped.

> Apologies about this mistake, which has been fixed (p. 22 of the revised manuscript).

9. The authors claim that "the procentriole likewise harbors little SAS-4 initially and that more protein is recruited at prometaphase, resulting in similar levels of SAS-4 in the centriole and the procentriole by then (Fig. 2D)". Can the authors provide some sort of semi-quantitative readout?

> Prompted by this suggestion, we quantified the amount of SAS-4 in 12 pairs of centrioles on individual spindle poles during mitosis. Importantly, we found the difference to be negligible (~14% on average, compared to ~1100% in interphase, see Figure 2A). This new quantification is reported in the in the legend of Figure 2 (p. 18 of the revised manuscript).

10. In Figure 5A side view, the presence of an inner tube is not very clear. Given that diameter quantifications were done using the mostly side views, it would be beneficial if the authors could provide a clearer image.

> The side view in Figure 5A was chosen because it displays most of the ultrastructural features discussed in the text in a favorable manner. However, we agree that this is not the most telling image for the inner tube. To address this issue, we have gone through the entire EM data set anew and have added a new Figure S3A that shows the inner tube in a clearer fashion. Moreover, we have curated the EM data to remove from the data set those side views in which inner tube diameter appears uncertain (going from N=44 to N=31), without impacting the resulting average diameter (see Figure %B, 5C).

Reviewer #2 (Significance (Required)):

Overall, these observations contribute toward a better understanding of centriole structure, molecular composition and diversity, with a particular focus on C. elegans. The precision of the approach developed by the authors (U-ExM and EM overlay) is a valuable tool and will be of interest to the centriole biology field and to cell biologists in general.
Reviewer expertise: Cellular and molecular biologists working in the field of centrioles.

Reviewer #3 (Evidence, reproducibility and clarity (Required)):

In this manuscript Woglar et al. use several light and electron microscopy techniques combined with averaging/registration methodologies to produce a comprehensive molecular map of the centriole in the C. elegans gonad. The images produced are very impressive and potentially very informative, allowing the authors to draw several important conclusions (e.g. about the chirality of the structure, and the potential organisation of Sas-6 in the cartwheel, the latter of which has been controversial in this species). Thus, although the manuscript is largely descriptive, there is a lot here that will be of great interest to the centriole field. The manuscript is generally well written and well presented, and, although I am not a great expert in all of these techniques, the data seems to solidly support the main conclusions. I therefore have only a small number of relatively minor suggestions for improvements.

Minor Comments:

1. It should be clarified whether the centrioles being examined here are organising genuine PCM and MTs. I know that in the embryo SPD-2 and SPD-5 are considered the main organisers of the mitotic PCM, and these centrioles are in S-phase or G2 (so I'm not sure if they are organising any PCM). SPD-5 is located internally to SPD-2, perhaps suggesting that these centrioles are not organising a bona fide PCM? On the other hand, TBG-1 and MZT-1 are located at the periphery, so I assume these centrioles are organising MTs?

> In the revised manuscript, we have referenced previous work establishing that centrioles during meiotic prophase do not organize PCM and do not act as microtubule organizing centers (MTOCs) (Zhou et al., 2009; doi: 10.1083/jcb.200902101; Mikeladze-Dvali et al., 2012; doi: 10.1242/dev.075440) (p. 17 of the revised manuscript).

2. I think the labels (A, B, C) in Figure S1 are probably in the wrong order and are not referred to correctly in the main text.

> Apologies about this mistake, which has been fixed (p. 22 of the revised manuscript).

3. In Figure S1A two centrioles are shown that seem to be touching at their proximal ends, which I initially interpreted as meaning the centrioles were engaged. If so, there seems to be a long tail

of Sas-6 connecting the two centrioles that extends well below the centriole MTs. However, reading the legend, I think this interpretation is incorrect, and the images are showing two separate centrioles that just happen to be touching? Perhaps swap in another image that won't lead to this potential confusion?

> Please see point 4 of reviewer #2.

Reviewer #3 (Significance (Required)):

Although several papers have reported high resolution molecular mapping of centrioles, this one is perhaps the most detailed and does a nice job of superimposing the molecular structures on high quality EM images. Not all of these C. elegans proteins are obviously conserved, but C. elegans is a 'poster-child' model organism for centriole research, and this broad architecture will be of great interest to the entire centriole/centrosome (and also cilia) fields. In addition, the observation of chirality that is intrinsic to the inner centriole structure, and that Sas-6 is likely organised into rings rather than a steep helix, are important conclusions.

I am an expert in centrioles and high resolution imaging, but not EM.
