# Supplementary material for: Molecular architecture of the C. elegans centriole
Source: PLoS Biol. 2022 Sep 15;20(9):e3001784. doi: 10.1371/journal.pbio.3001784 (PMC9531800; doi:10.1371/journal.pbio.3001784)
Supplement: Peer review history

# Author comment: Author response to previous submission

**DOI:** 10.1371/journal.pbio.3001784.r001

1 Jul 2022

Attachment Submitted filename: 126915_1_rebuttal_2397858_r777x3.pdf Click here for additional data file.

# Editor report: Decision Letter 0

**DOI:** 10.1371/journal.pbio.3001784.r002

**Authors:** Ines Alvarez-Garcia

12 Jul 2022

Dear Dr Gönczy, 

Thank you for submitting your Review Commons manuscript entitled "Molecular architecture of the C. elegans centriole" for consideration as a Research Article by PLOS Biology.

Your manuscript and associated reviews have now been evaluated by the PLOS Biology editorial staff as well as by an academic editor with relevant expertise and I am writing to let you know that we would like to consider the manuscript for publication pending the revisions you have stated in the rebuttal.

However, before we can send you the decision, we need you to complete your submission by providing the metadata that is required to consider the paper further for publication. To this end, please login to Editorial Manager where you will find the paper in the 'Submissions Needing Revisions' folder on your homepage. Please click 'Revise Submission' from the Action Links and complete all additional questions in the submission questionnaire.

Once your full submission is complete, your paper will undergo a series of checks to make sure we have all the data we need. To provide the metadata for your submission, please Login to Editorial Manager (https://www.editorialmanager.com/pbiology) within two working days, i.e. by Jul 14 2022 11:59PM.

During the process of completing your manuscript submission, you will be invited to opt-in to posting your pre-review manuscript as a bioRxiv preprint. Visit http://journals.plos.org/plosbiology/s/preprints for full details. If you consent to posting your current manuscript as a preprint, please upload a single Preprint PDF. 

Feel free to email us at plosbiology@plos.org if you have any queries relating to your submission.

Kind regards,

Ines

--

Ines Alvarez-Garcia, PhD

Senior Editor

PLOS Biology

ialvarez-garcia@plos.org

# Author comment: Author response to Decision Letter 0

**DOI:** 10.1371/journal.pbio.3001784.r003

25 Jul 2022

Attachment Submitted filename: Woglar_2022_response_to _reviewers.pdf Click here for additional data file.

# Editor report: Decision Letter 1

**DOI:** 10.1371/journal.pbio.3001784.r004

**Authors:** Ines Alvarez-Garcia

4 Aug 2022

Dear Dr Gönczy,

Thank you for the submission of your revised Research Article entitled "Molecular architecture of the C. elegans centriole" for publication in PLOS Biology. On behalf of my colleagues and the Academic Editor, Renata Basto, I am happy to say that we can in principle accept your manuscript for publication, provided you address any remaining formatting and reporting issues. These will be detailed in an email you should receive within 2-3 business days from our colleagues in the journal operations team; no action is required from you until then. Please note that we will not be able to formally accept your manuscript and schedule it for publication until you have completed any requested changes.

Please take a minute to log into Editorial Manager at http://www.editorialmanager.com/pbiology/, click the "Update My Information" link at the top of the page, and update your user information to ensure an efficient production process.

PRESS

We frequently collaborate with press offices. If your institution or institutions have a press office, please notify them about your upcoming paper at this point, to enable them to help maximise its impact. If the press office is planning to promote your findings, we would be grateful if they could coordinate with biologypress@plos.org. If you have previously opted in to the early version process, we ask that you notify us immediately of any press plans so that we may opt out on your behalf.

We also ask that you take this opportunity to read our Embargo Policy regarding the discussion, promotion and media coverage of work that is yet to be published by PLOS. As your manuscript is not yet published, it is bound by the conditions of our Embargo Policy. Please be aware that this policy is in place both to ensure that any press coverage of your article is fully substantiated and to provide a direct link between such coverage and the published work. For full details of our Embargo Policy, please visit http://www.plos.org/about/media-inquiries/embargo-policy/.

Many congratulations and thanks again for choosing PLOS Biology for publication and supporting Open Access publishing. We look forward to publishing your study. 

Sincerely, 

Ines

--

Ines Alvarez-Garcia, PhD

Senior Editor

PLOS Biology

ialvarez-garcia@plos.org
